# Non-Genetic Risk Factors of Alzheimer's Disease: An Updated Umbrella Review

**DOI:** 10.14283/jpad.2024.100

**Published:** 2024-05-29

**Authors:** S.-Y. He, W.-M. Su, X.-J. Wen, S.-J. Lu, B. Cao, Bo Yan, Yong-Ping Chen

**Affiliations:** 1 Department of Neurology, West China Hospital, Sichuan University, 610041, Chengdu, Sichuan, China; 2 West China School of Medicine, West China Hospital, Sichuan University, 610041, Chengdu, Sichuan, China; 3 Department of Respiratory, The Fourth People's Hospital of Chengdu, Mental Health Center of Chengdu, 610036, Chengdu, Sichuan, China

**Keywords:** Alzheimer's disease, modifiable risk factors, meta-analysis, umbrella review

## Abstract

**Background:**

Alzheimer's disease (AD) is a progressive neurodegenerative disorder characterized by intricate genetic and environmental etiology. The objective of this study was to identify robust non-genetic risk factors for AD through an updated umbrella review.

**Methods:**

We conducted a comprehensive search of meta-analyses and systematic reviews on non-genetic risk factors associated with AD in PubMed, Cochrane, Embase, and Ovid Medline up to June 30, 2023. After collecting data, we estimated the summary effect size and their 95% confidence intervals. The degree of heterogeneity between studies was assessed using I^2^ statistics and a 95% prediction interval was determined. Additionally, we evaluated potential excess significant bias and small study effects within the selected candidate studies.

**Results:**

The umbrella review encompassed a total of 53 eligible papers, which included 84 meta-analyses covering various factors such as lifestyle, diet, environmental exposures, comorbidity or infections, drugs, and biomarkers. Based on the evidence classification criteria employed in this study, two factors as convincing evidence (Class I), including rheumatoid arthritis (RA), potentially reduced the risk of AD, but diabetes significantly increased the risk of AD. Furthermore, three factors as highly suggestive evidence (Class II), namely depression, high homocysteine, and low folic acid level, potentially increased the risk of AD.

**Conclusion:**

Our findings highlight several risk factors associated with AD that warrant consideration as potential targets for intervention. However, it is crucial to prioritize the identified modifiable risk factors, namely rheumatoid arthritis, diabetes, depression, elevated homocysteine levels, and low folic acid levels to effectively address this complex neurodegenerative disorder.

## Introduction

**A**lzheimer's disease (AD) is an insidious and irreversible neurodegenerative disorder, which represents the main etiology of dementia clinically characterized by memory loss, cognitive decline, behavior changes, and functional impairment (1). Currently, the prevalence of AD is 4%∼7% in the global elderly population over 65 years old, and the prevalence of AD can be as high as 20%∼30% in the elderly population over 85 years old (2). Based on the 2020 Alzheimer's disease facts and figures, the number of AD patients in the United States could increase significantly from 5.8 million to 13.8 million by 2050. Over the past few decades, community surveys in Japan and China have found a significant increase in the prevalence of AD (3). Consequently, this exacerbates both the economic burden on AD patients and their families' caregiving responsibilities (4).

The etiology of AD is multifaceted and intricate, with the main pathologic features of amyloid plaque formation and neurofibrillary tangles (5, 6). While familial AD accounts for a portion of patients, approximately one-third of AD cases may be attributed to non-genetic modifiable factors such as cardiovascular diseases, diabetes, smoking, alcohol consumption, physical activity, and so on (6, 7). Given that there is currently no effective treatment for AD, it is imperative to identify the risk factors associated with AD and address them in order to prevent or delay the onset of this debilitating condition. In Western countries, the prevalence and incidence of dementia appear to be declining due to insights gained from several population-based longitudinal studies focusing on modifiable factors (8, 9, 10, 11, 12). For instance, randomized clinical trials have demonstrated that engaging in regular physical activity can enhance cognitive function and mitigate the rate of cognitive decline (13), shedding light on a potential association between physical activity and AD. However, it is crucial for clinical trials to consider the characteristics of the included population as well as employ long-term follow-up strategies given the slow progression nature of AD. Furthermore, most trials primarily focus on assessing cognitive decline rather than disease progression. To address this matter comprehensively, numerous systematic reviews and meta-analyses have been conducted to analyze the risk factors associated with AD (14, 15, 16). Nevertheless, these studies failed to offer a comprehensive evaluation and consistent recommendations regarding the risk factors associated with AD.

Umbrella reviews, which systematically evaluate meta-analyses and/or systematic reviews, encompass multiple comparisons within the same disease and possess a higher level of evidence classification. Therefore, we selected this approach to summarize and update non-genetic risk factors associated with AD based on established guidelines (17), aiming to arrive at a conclusion by integrating all available meta-analyses and systematic reviews. Subsequently, robust associations between these factors and AD were also identified.

## Methods

The protocol for this study was preregistered in the International Prospective Register of Systematic Reviews (PROSPERO) under the ID number CRD42023454097. This umbrella review was conducted in accordance with the guidelines (17) and followed the updated Preferred Reporting Items for Systematic Reviews and Meta-analysis (18).

### Search strategy

We conducted a comprehensive search on PubMed, Cochrane, Embase, and Ovid Medline up to June 30, 2023, in order to identify meta-analyses and systematic reviews of observational studies investigating the associations between potential biomarkers or environmental risk factors for AD. Our search strategy employed the terms (Alzheimer* OR dementia) AND (meta-analyses OR systematic review) AND risk, with each term limited to abstracts or titles. Two independent investigators (SYH and WMS) were responsible for meticulously reviewing the full text of potentially eligible articles.

### Eligibility criteria

We included meta-analyses of observational studies investigating risk factors associated with AD, including prospective cohort studies, case-control studies, and cross-sectional studies. Firstly, these meta-analyses were required to report the effect size, 95% confidence interval (95% CI), and any potential publication bias either in the text or graph. Secondly, only one meta-analysis with the largest number of primary studies or the most recent update was selected for each exposure to avoid duplicate studies concerning the same factor.

If the articles exclusively investigated non-modifiable genetic factors associated with AD, they were excluded. Meta-analyses solely focusing on imaging, diagnostic, prognostic, and other non-observational studies were also excluded. Additionally, articles that primarily examined outcomes related to mild cognitive impairment, cognitive impairment severity, and symptom progression of AD rather than AD risk were excluded as well. Lastly, articles where results for AD could not be extracted from subgroups in patients with dementia were also excluded.

### Quality assessment and data extraction

The methodological quality of the included articles was assessed using the Measurement Tool for Assessing Systematic Reviews (AMSTAR), a valid and reliable measurement tool for assessing the quality of systematic reviews and meta-analyses (19). Two reviewers (SYH and WMS) conducted the assessment. The included studies were categorized as high quality (scores 8–11), medium quality (scores 4–7), or low quality (scores 0–3) (20). Subsequently, we employed the Grading of Recommendations, Assessment, Development and Evaluation (GRADE) framework to evaluate the quality of evidence (21), which classified it as “high”, “moderate”, “low”, or “very low”.

Two independent reviewers conducted the study selection and data extraction processes. Relevant information, such as first author, publication year, factors studied, and number of AD cases were extracted from eligible meta-analyses and systematic reviews. Additionally, we retrieved risk estimates such as odds ratio (OR), hazard ratio (HR), and risk ratio (RR), along with their corresponding 95%CI for each study. Quality assessment of the studies was noted when available. In cases where multiple control groups were used, data from healthy groups took priority.

### Statistical analysis

The pooled effect size and the 95%CI were estimated for each meta-analysis using both random-effects and fixed-effects models (22). In cases involving continuous data, the standard mean difference (SMD) was converted to an odds ratio (OR) by multiplying it by π/√3 (23, 24). Additionally, a 95% prediction interval (PI), accounting for variation in different settings, was calculated (25, 26). If the 95% PI crosses the null value, it indicates that these factors may not have an effect or even have an opposite effect. To estimate OR, RR, and HR accurately, natural logarithm scale transformations were applied. For calculation purposes, the standard error (SE) of the pooled effect size (M) and the heterogeneity estimate (τ^2^) are required. The 95% PI is calculated as log M ± t_1-0.05/2, k-1_ × √(τ^2^+SE^2^) (26). An approximation was made for the SE of the effect size in the largest study of each meta-analysis. If SE < 0.10, we evaluated whether the difference between the effect estimation and the lower or upper 95% CI was less than 0.20 to ascertain the presence of a small study effect. Heterogeneity among studies was quantified using the I^2^ metric (27). I^2^ ranges from 0% to 100% and represents the proportion of variation in effect estimation due to heterogeneity rather than sampling error, which is commonly calculated in most published meta-analyses (28). I^2^ > 50% or > 75% indicates large or very large heterogeneity, respectively.

The presence of small study effects, which may contribute to publication bias, was assessed using Egger's test and visualized on a funnel plot. A p-value greater than 0.10 indicated that the degree of asymmetry was small and non-significant (29, 30). Conversely, it indicated the presence of small study effects, particularly in larger studies with more conservative effects. Additionally, we employed the excess statistical significance test to assess if the number of observed studies (O) with positive results (p < 0.05) exceeded the expected number (E) (31). By utilizing effect size, 95% CI, and standard error of studies in each meta-analysis, we calculated each study power through a power-enhanced funnel plot (31, 32). When a two-tailed p-value was less than 0.10 and O surpassed E, it indicated an excess of observed studies compared to what was expected.

### Assessment of Epidemiological Credibility

Based on the classification of evidence from previous studies (33, 34), we categorized the identified factors into five distinct classes: Class I (convincing evidence), Class II (highly suggestive evidence), Class III (suggestive evidence), Class IV (weak evidence), and NS (nonsignificant). Detailed criteria for the classification of evidence are presented in Table 2.

**Table 2:** Summary of 84 meta-analysis about risk factors with Alzheimer's disease

Classification	Definition	Description	Risk factors
Class I	Convincing evidence	cases >1000, p < 10^−6^ at random-effects model, 95% prediction interval excluded the null value, no large heterogeneity (I^2^ < 50%) and no small study effects or excess significant bias	RA, diabetes
Class II	Highly suggestive evidence	cases >1000, p < 10^−6^ at random-effects model, 95% CI of the largest study excluded the null value	Depression, high homocysteine, and low folic acid level
Class III	Suggestive evidence	cases > 1000, p < 10^−3^ at random-effects model	Atrial fibrillation, vitamin E in the diet, atherosclerosis, exposure to aluminum, leisure activities, statin, bone loss, head injury, epilepsy, midlife hypertension, AHMs, all NSAIDs, aspirin, Cu in serum, extremely low-frequency magnetic fields, plasma/serum zeaxanthin, sleep disturbances, ApoA-I in serum, no-aspirin NSAIDs, cataract, and antioxidants
Class IV	Weak evidence	p < 0.05	Current versus never smoking, pesticide, alcohol consumption, ApoA-I in plasma, TC, clusterin in CSF, headache, BMD, hearing loss, albuminuria, HSV-1, CMV, Cpn, cancer, serum uric acid, VD deficiency, long sleep, plasma/serum lutein levels, low testosterone, AMD, tooth loss, HDP, stroke, vitamin C, cognitive activity, and loneliness
NS	Non-significant evidence	p > 0.05	Ever versus never smoking, CHD, heart failure, socioeconomic status, ApoA-I in CSF, HDL-C, LDL-C, TG, clusterin in plasma, underweight, overweight, obese, caffeine, frailty, peripheral blood BDNF, late-life hypertension, metformin, IBD, Zn in serum, Fe in serum, GA, short sleep, PM2.5, O3, plasma/ serum β-carotene levels, plasm/serum lycopene, plasma/serum α-carotene levels, plasma/serum β-cryptoxanthin, DHA, EPA, PPI, and high education


TC: total cholesterol, TG: total triglycerides, HDL-C: high-density lipoprotein, LDL-C: low-density lipoprotein, CHD: coronary heart disease, ApoA-I: Napoli protein A1, BMI: body mass index, RA: rheumatoid arthritis, BMD: bone mineral density, HSV-1: herpes simplex virus, CMV: cytomegalovirus, Cpn: chlamydia pneumonia, AHMs: anti-hypertensive medications, NSAIDs: non-steroidal anti-inflammatory drugs, IBD: inflammatory bowel disease, VD: vitamin D, GA: general anesthesia, PM2.5: particulate matter, O3: ozone, DHA: docosahexaenoic acid, EPA: eicosapentaenoic acid, AMD: age-related macular degeneration, PPI: proton pump inhibitors, HDP: hypertensive disorders of pregnancy

Although certain associations were supported as Class I or Class II evidence, it is imperative to acknowledge that this does not establish a causal relationship between risk factors and the disease. Therefore, we must meticulously scrutinize these two levels of evidence. The statistical analysis and the power evaluation were conducted utilizing Stata 17.0 and R 4.3.1.

## Results

### Search results

**Figure 1:**
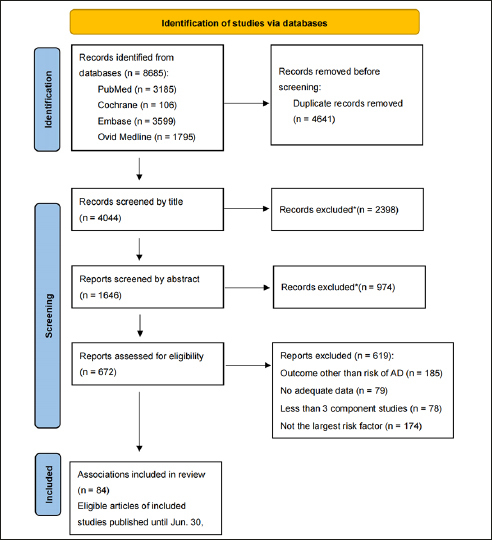
Flowchart of literature research *When articles are screened by title and abstract, they are excluded because of non-observational studies, non-human studies, genetic studies, or studies' outcome other than risk of AD.

After conducting an extensive search of a total of 8684 articles, we identified 53 articles that met our criteria (Fig. 1). These 53 articles encompassed a cumulative total of 84 meta-analyses, which included 892 observational studies and were published between 2013 and 2023. The 84 meta-analyses examined various types of risk factors, including habits (9), dietary factors (6), environmental exposures (8), diseases (26), drugs (7), and biomarkers (28). Notably, among these meta-analyses, 60 had sample sizes exceeding 1000 cases (Table 1). Importantly, the eligible meta-analyses did not have direct access to individual participant data; instead, they solely relied on summary-level information derived from published research.

**Table 1:** Quantitative synthesis of 84 meta-analyses in 53 eligible articles investigating risk factors with Alzheimer's disease

Reference	Risk factor	Number of cases	Number of studies	Effect size	Random-effects summary effect size(95% CI)	P random	95% PI	I^2^	Small study effects/excess significance bias	Classification
**Habits**										
Zhong, 2015[88]	Ever versus never smoking	5816	22	RR	1.12(0.99–1.26)	6.50E-02	0.56–5.16	55.90%	Yes**/No**	NS
Zhong, 2015[88]	Current versus never smoking	4772	12	OR	1.41(1.11–1.80)	5.30E-03	0.61–3.23	66.80%	No/No	IV
Xie, 2022[16]	Alcohol consumption	2321	12	RR	0.67(0.50–0.92)	0.0122	0.22–2.07	88.30%	No/No	IV
Su, 2022[89]	Leisure activities	2848	15	RR	0.82(0.74–0.91)	0.0001	0.55–1.23	69.70%	Yes/No	III
Kim, 2015[90]	Caffeine	590	5	OR	0.79(0.49–1.27)	0.3311	0.18–3.43	71.00%	No/No	NS
Fan, 2019[54]	Long sleep	NR	6	HR	1.62(1.19–2.21)	2.40E-03	0.74–3.53	44.40%	No/No	IV
Fan, 2019[54]	Short sleep	NR	6	HR	1.19(0.91–1.56)	2.10E-01	0.58–2.42	57.80%	No/No	NS
Yu, 2020[60]	Cognitive activity	532	6	RR	0.49(0.38–0.63)	2.36E-08	0.27–0.89	35.8%	No/No	IV
Qiao, 2022[91]	Loneliness	411	3	RR	1.74(1.30–2.34)	2.00E-04	0.77–3.91	17.80%	NE/No	IV
Dietary factors										
Zhao, 2022[92]	Vitamin E in the diet	5409	9	OR	0.77(0.68–0.87)	0.000023	0.67–0.88	36.80%	No/No	III
Chai, 2019[38]	VD deficiency	14618	6	HR	1.36(1.13–1.65)	1.30E-03	0.79–2.35	53.20%	No/No	IV
Zhu, 2021[48]	DHA	1560	3	RR	0.75(0.42–1.34)	3.40E-01	0.08–7.35	76.20%	NE/No	NS
Zhu, 2021[48]	EPA	1560	3	RR	0.91(0.66–1.25)	5.60E-01	0.29–2.86	64.80%	NE/No	NS
Zhao, 2023[93]	Antioxidants	34769	12	RR	0.83(0.74–0.92)	7.00E-04	0.53–1.31	45.70%	Yes/No	III
Yu, 2020[60]	Vitamin C	978	6	RR	0.84(0.71–1.00)	4.62E-02	0.67–1.06	0.00%	No/No	IV
Disease or infection										
Zuin, 2021[94]	Atrial fibrillation	60785	9	HR	1.30(1.11–1.47)	1.00E-04	0.87–1.95	85.20%	No/No	III
Xie, 2020[95]	Atherosclerosis	1698	10	OR	1.50(1.24–1.80)	1.91E-05	0.72–3.13	88.20%	Yes/No	III
Wolters, 2018[14]	CHD	>1000*	8	RR	1.09(0.90–1.32)	0.38	0.72–1.65	30.20%	Yes/No	NS
Wolters, 2018[14]	Heart failure	>1000*	5	RR	1.41(0.98–2.03)	0.61	0.52–3.82	73.80%	Yes/No	NS
Qu, 2022[96]	Headache	>1000*	7	OR	1.53(1.06–2.22)	0.0248	0.52–4.53	70.40%	No/No	IV
Liang 2021[97]	Hearing loss	>1000*	5	HR	2.23(1.33–3.73)	2.30E-03	1.084.59	1.60%	No/No	IV
Li, 2017[98]	Head injury	8166	28	RR	1.51(1.27–1.80)	3.24E-06	0.63–3.60	70.10%	No/No	III
Policicchio, 2017[57]	RA	1037	10	OR	0.60(0.49–0.72)	1.06E-07	0.47–0.76	33.20%	No/No	I
Mehta, 2022[58]	Depression	>1000*	27	OR	1.79(1.46–2.20)	1.71E-08	0.68–4.74	94.50%	No/Yes	II
Mehta, 2022[58]	Bone loss	>1000*	3	OR	1.81(1.28–2.55)	7.00E-04	0.56–5.84	43.80%	NE/No	III
Dun, 2022[99]	Epilepsy	63261	5	HR	2.24(1.39–3.59)	9.00E-04	0.59–8.56	78.10%	No/No	III
Zhang, 2022[100]	Cancer	>1000*	15	RR	0.86(0.78–0.94)	2.20E-03	0.61–1.21	91.90%	No/No	IV
Ou, 2020[63]	HSV-1	814	18	OR	1.34(1.02–1.75)	3.34E-02	1.02–1.76	0.00%	No /Yes	IV
Ou, 2020[63]	CMV	449	8	OR	1.39(1.05–1.83)	1.97E-02	0.99–1.93	12.70%	No/No	IV
Ou, 2020[63]	Cpn	389	11	OR	4.56(1.59–13.05)	4.60E-03	0.19–111.21	71.40%	No/No	IV
Ou, 2020[61]	Midlife hypertension	2279	4	RR	1.19(1.08–1.32)	7.00E-04	1.02–1.40	0.00%	No/No	III
Ou, 2020[61]	Late-life hypertension	4251	18	RR	0.94(0.85–1.05)	2.60E-01	0.75–1.18	24.70%	Yes/Yes	NS
Gudala, 2013[51]	Diabetes	4592	20	RR	1.58(1.43–1.76)	7.18E-18	1.07–1.94	13.30%	No/No	I
Shi, 2018[49]	Sleep disturbances	4627	*9*	RR	1.70(1.24–2.33)	1.10E-03	0.56–5.16	75.70%	No/No	III
Liu, 2022[36]	IBD	NR	*5*	RR	1.65(0.84–3.26)	1.40E-01	0.16–16.62	99.20%	Yes/No	NS
Kojima, 2016[101]	Frailty	>1000*	4	HR	1.28(0.88–1.85)	1.94E-01	0.47–3.48	51.10%	No/No	NS
Tsai, 2023[52]	AMD	84060	6	HR	1.21(1.02–1.44)	3.00E-02	0.73–1.99	69.30%	Yes/No	IV
Li, 2023[102]	Tooth loss	NR	6	RR	1.11(1.03–1.20)	6.00E-03	0.87–1.41	61.40%	Yes/No	IV
Xiong, 2023 [53]	Cataract	>1000	*9*	HR	1.17(1.08–1.27)	7.97E-05	0.96–1.43	48.80%	No/No	III
Schliep, 2023[39]	HDP	>1000	3	HR	1.40(1.13–1.74)	1.80E-03	0.87–2.25	14.00%	NE/No	IV
Yu, 2020[60]	Stroke	1732	7	RR	1.38(1.02–1.88)	3.00E-02	0.70–2.74	28.00%	No/No	IV
Exposure to environment										
Yu, 2020[60]	High education	1727	7	RR	0.95(0.89–1.00)	0.057	0.79–1.14	74.2%	No/No	NS
Yan, 2016[62]	Pesticide	1050	7	OR	1.34(1.08–1.67)	0.0076	1.02–1.75	0.00%	No/No	IV
Wang, 2016[103]	Exposure to aluminum	1383	8	OR	1.72(1.33–2.21)	2.79E-05	1.19–2.49	6.20%	No/No	III
Wang, 2022[104]	Socioeconomic status	7276	5	RR	1.19(0.70–2.03)	0.5103	0.22–6.53	83.30%	Yes/No	NS
Lee, 2020[42]	General anesthesia	NR	17	OR	0.92(0.82–1.05)	2.40E-01	0.73–1.16	20.90%	No/No	NS
Jalilian, 2018[43]	Extremely low-frequency magnetic fields	7362	20	RR	1.72(1.37–2.15)	2.10E-06	0.69–4.28	61.00%	No/No	III
Dhiman, 2022[44]	PM2.5	10633	6	HR	1.08(0.98–1.18)	1.00E-01	0.82–1.42	99.20%	No/No	NS
Dhiman, 2022[44]	O3	10165	4	HR	1.02(0.97–1.07)	4.50E-01	0.84–1.23	99.50%	No/No	NS
Biomarkers										
Tong, 2022[105]	ApoA-I in plasma	246	5	OR	0.12(0.02–0.67)	0.015	0.004–3.21	94.40%	No/No	IV
Tong, 2022[105]	ApoA-I in serum	747	9	OR	0.12(0.04–0.34)	6.47E-05	0.01–1.5	95.90%	No/No	IV
Tong, 2022[105]	ApoA-I in CSF	201	5	OR	1.43(0.74–2.76)	2.70E-01	0.48–4.24	65.30%	No/No	NS
Tang, 2019[106]	TC	2159	25	OR	1.35(1.02–1.78)	3.00E-02	0.66–2.74	82%	No/Yes	IV
Tang, 2019[106]	HDL-C	1671	18	OR	0.76(0.54–1.1)	1.50E-01	0.32–1.77	87%	No/Yes	NS
Tang, 2019[106]	LDL-C	1627	17	OR	1.39(1.03–2.00)	8.00E-02	0.60–3.21	87%	No/No	NS
Tang, 2019[106]	TG	1589	17	OR	1.29(1.12–1.85)	1.70E-01	0.56–2.97	87%	No/Yes	NS
Shi, 2019[59]	Clusterin in plasma	1871	13	OR	1.41(1.19–2.39)	0.2	0.45–4.4	93%	No/No	NS
Shi, 2019[59]	Clusterin in CSF	342	3	OR	2.7(2.13–3.65)	8.16E-14	0.35–20.37	0%	NE/No	IV
Rahmani, 2022[15]	Underweight	>1000*	7	HR	1.43(0.86–2.39)	0.1702	0.30–6.75	60.40%	No/No	NS
Rahmani, 2022[15]	Overweight	>1000*	8	HR	1.02(0.80–1.30)	0.8724	0.46–2.26	87.40%	No/No	NS
Rahmani, 2022[15]	Obese	>1000*	6	HR	1.20(0.76–1.88)	0.4312	0.27–5.23	81.90%	No/No	NS
Lv, 2018[107]	BMD	310	4	OR	0.11(0.03–0.38)	5.34E-04	0.004–2.45	95.90%	NE/No	IV
Li, 2022[108]	Albuminuria	741	6	OR	1.37(1.05–1.79)	2.04E-02	0.77–2.44	17.40%	Yes/No	IV
Kim, 2017[109]	Peripheral blood BDNF	1455	20	OR	0.74(0.48–1.15)	0.18	0.98–1.44	85.80%	No/Yes	NS
Qu, 2021[41]	Plasma/Serum β-carotene levels	1155	14	OR	0.96(0.52–1.80)	9.10E-01	0.22–4.14	94.40%	No/No	NS
Qu, 2021[41]	Plasma/Serum α-carotene levels	889	10	OR	0.69(0.32–1.49)	3.40E-01	0.13–3.63	95.50%	No/Yes	NS
Qu, 2021[41]	Plasma/Serum lycopene	883	10	OR	0.80(0.15–4.29)	8.00E-01	0.01–35.49	99.10%	No/Yes	NS
Qu, 2021[41]	Plasma/Serum lutein levels	846	10	OR	3.81(1.97–7.39)	6.89E-05	0.92–15.64	92.10%	No/No	IV
Qu, 2021[41]	Plasma/Serum β-cryptoxanthin	860	9	OR	0.85(0.21–3.46)	8.20E-01	0.73–11.91	98.70%	No/Yes	NS
Qu, 2021[41]	Plasma / Serum zeaxanthin	846	10	OR	2.95(1.53–5.59)	1.00E-03	0.16–3.77	92.00%	No/No	III
Du, 2016[56]	Serum uric acid	1128	20	OR	3.86(1.48–10.05)	6.00E-03	0.29–51.22	97.10%	No/Yes	IV
Li, 2017[37]	Cu in serum	2128	35	OR	3.36(2.03–5.58)	2.66E-06	0.64–17.50	94.40%	No/No	III
Li, 2017[37]	Zn in serum	1027	22	OR	0.62(0.30–1.27)	1.90E-01	0.08–4.37	94.70%	No/Yes	NS
Li, 2017[37]	Fe in serum	1379	25	OR	0.81(0.40–1.67)	6.00E-01	0.10–6.36	95.70%	No/Yes	NS
Shen, 2015[55]	High homocysteine level	4830	9	RR	1.89(1.54–2.33)	1.67E-09	1.11–2.81	0.00%	Yes/No	II
Shen, 2015[55]	Low folic acid level	2070	6	RR	2.22(1.71–2.89)	2.73E-09	0.95–4.68	0.00%	No/No	II
Lv, 2016[50]	Low testosterone	240	7	RR	1.49(1.12–1.98)	6.10E-03	0.74–3.00	47.20%	No/No	IV
Drugs										
Poly, 2020[110]	Statin	>1000*	20	RR	0.70(0.60–0.80)	1.27E-06	0.45–2.08	47.90%	Yes/No	III
Ou, 2020[61]	AHMs	3424	12	RR	0.81(0.72–0.91)	2.00E-04	0.59–1.10	52.10%	No/No	III
Wang, 2015[111]	All NSAIDs	13407	16	RR	0.70(0.57–0.85)	3.00E-04	0.34–1.43	75.00%	No/No	III
Wang, 2015[111]	Aspirin	14303	11	RR	0.77(0.64–0.93)	5.80E-03	0.45–1.33	52.10%	No/No	III
Wang, 2015[111]	No-aspirin NSAIDs	29393	8	RR	0.65(0.48–0.88)	4.90E-03	0.29–1.47	63.40%	No/No	III
Luo, 2022[35]	Metformin	>1000*	10	OR	1.15(0.82–1.63)	4.03E-01	0.28–4.70	94.20%	No/No	NS
Ahn, 2023[45]	PPI	106491	5	RR	1.15(0.95–1.40)	1.60E-01	0.62–2.13	96.90%	No/No	NS


For biomarkers, ApoA-I and clusterin were classified by plasma and CSF respectively. Fat, carotenoid, homocysteine, folic acid, and metals such as Cu, Zn, and Fe were analyzed from low to high levels. Other evidence including drugs, exposure, and diseases were mostly analyzed by yes and no. TC: total cholesterol, TG: total triglycerides, HDL-C: high-density lipoprotein, LDL-C: low-density lipoprotein, CHD: coronary heart disease, ApoA-I: Napoli protein A1, BMI: body mass index, RA: rheumatoid arthritis, BMD: bone mineral density, HSV-1: herpes simplex virus, CMV: cytomegalovirus, Cpn: chlamydia pneumonia, AHMs: anti-hypertensive medications, NSAIDs: non-steroidal anti-inflammatory drugs, IBD: inflammatory bowel disease, VD: vitamin D, GA: general anesthesia, PM2.5: particulate matter, O3: ozone, DHA: docosahexaenoic acid, EPA: eicosapentaenoic acid, AMD: age-related macular degeneration, PPI: proton pump inhibitors, HDP: hypertensive disorders of pregnancy, HR: hazard ratio, OR: odds ratio, RR: relative risk, PI: prediction interval. *For some meta-analyses, the authors conducted associations with AD and dementia, We can only roughly judge that the sample size may be greater than 1000.

### Quality assessment

Among the 53 articles, a total of 34 articles employed the Newcastle-Ottawa Scale (NOS) for qualitative assessment, encompassing 496 studies. Out of these studies, 70 (14%) were classified as high quality (NOS score = 9), while moderate quality was observed in 270 (54%) studies with NOS scores ranging from 7 to 8. Additionally, low quality was identified in 156 (32%) studies with NOS scores below 7. Five articles (35–39) utilized both the NOS and either the Agency for Healthcare Research (40) and Quality or the Cochrane Risk of Bias tool for assessment purposes. Furthermore, five articles (41, 42, 43, 44, 45) employed alternative evaluation tools such as the Hill Criteria (46) and open-access Joanna Briggs Institute criteria (47) among others. Moreover, seven articles (48, 49, 50, 51, 52, 53, 54) did not provide sufficient data on quality assessment, and two publications (55, 56) refrained from utilizing any assessment tool due to potential bias concerns (Supplementary Table 1).

### Characteristics and Quantitative Analysis

After summarizing these data, statistically significant effects (p < 0.05) were observed in 53 out of the 84 meta-analyses conducted using the random-effects model. Among these, 27 demonstrated highly significant effects at p < 0.001 (Table 2). Importantly, seven factors, namely rheumatoid arthritis (RA) (57), diabetes (51), depression (58), homocysteine (55), folic acid (55), clusterin in cerebrospinal fluid (59), and cognitive activity (60), exhibited remarkably significant effects at p < 10^−6^ (Table 1, Supplementary Table 2). Notably, among all associations examined, nine risk factors including RA (57), diabetes (51), homocysteine (55), vitamin E supplementation, midlife hypertension (61), extremely low-frequency magnetic fields (43), exposure to pesticide (62), HSV-1 infection (63), and testosterone levels (50) showed a strong association with AD as indicated by their respective 95% PI excluding the null value (Table 1).

### Bias assessment

Furthermore, among the 84 meta-analyses conducted, negligible heterogeneity estimates were observed in 27 (32%) cases, while moderate heterogeneity (I^2^ ≥ 50%, but < 75%) was exhibited in 22 (26%) cases and substantial heterogeneity (I^2^ > 75%) was displayed in 35 (42%) cases (Supplementary Table 3). Moreover, small study effects of evidence were observed in 14 (17%) meta-analyses, and excess significant bias was noted in 13 (15%) meta-analyses. However, for six (7%) meta-analyses, the presence of small study effects could not be estimated due to the inclusion of fewer than three studies.

### Quantitative Synthesis with Evidence Classification

As previously mentioned above, among the 84 meta-analyses, 7 (8%) demonstrated a significant association with AD at p < 10^−6^ under the random-effects model (Table 1, Supplementary Table 2). The median AMSTAR score for all evidence was 8.6 (range: 6–11). Detailed criteria and scores for the AMSTAR assessment are presented in Supplementary Table 4. Due to the inclusion of observational studies and factors that degrade quality, such as inconsistency, indirectness, imprecision, significant risk of bias, and publication bias, all evaluated evidence using GRADE was categorized as “low” or “very low” quality (Supplementary Table 5). After applying the evidence classification criteria to assess these risk factors, most of them were classified as Class III (21, 25%), IV (26, 31%), or NS (32, 38%) (Table 2, Supplementary Table 6). However, it is important to note five factors classified as Class I and Class II evidence (Table 3). Among these factors, RA and diabetes, were evaluated as having a “low” quality but demonstrated an association based on criteria including more than1000 cases, p < 10^−6^, no small study effects or excess significant bias, the 95% PI excluded null value, and non-significant heterogeneity. Additionally, the three other risk factors namely depression (low), homocysteine (very low), and folic acid (very low) were classified as Class II evidence due to meeting criteria such as having more than1000 cases, p < 10^−6^, and exhibiting the largest study with a 95% CI excluding null value.

**Table 3:** Quantitative synthesis of five associations with Alzheimer's disease with convincing or highly suggestive evidence

Reference	Risk factor	Number of cases	Number of primary studies	Effect size	Random-effects summary effect size(95%CI)	P random	95'%, PI	I_2_	Classification
Policicchio, 2017[57]	RA	1037	10	OR	0.60(0.49–0.72)	1.06E-07	0.47–0.76	33.20%	I
Gudala, 2013[51]	Diabetes	4592	20	RR	1.58(1.43–1.76)	7.18E-18	1.07–1.94	13.30%	I
Mehta, 2022[58]	Depression	>1000	27	OR	1.79(1.46–2.20)	1.71E-08	0.68–4.74	94.50%	II
Shen, 2015[55]	High Homocysteine level	4830	9	RR	1.89(1.54–2.33)	1.67E-09	1.11–2.81	0.00%	II
	Low Folic Acid level	2070	6	RR	2.22(1.71–2.89)	2.73E-09	0.95–4.68	0.00%	II


RA: rheumatoid arthritis, CHD: coronary heart disease; OR: odds ratio, RR: relative risk, PI: prediction interval

## Discussion

Here, we performed an umbrella review to establish a definitive correlation between biomarkers, environmental risk factors, and AD. Through meticulous quantitative synthesis and bias assessment, two factors pertaining to RA and diabetes were classified as Class I evidence for their associations with AD. Meanwhile, three factors (depression, elevated homocysteine levels and insufficient folic acid) were categorized as Class II evidence despite the presence of cases exceeding 1000 and p-values less than 10^−6^; however, it is worth noting that there may exist substantial heterogeneity in these studies along with a 95% PI encompassing the null hypothesis. Additionally, potential small-study effects and/or excess significance bias cannot be disregarded.

Two factors, RA and diabetes, presented Class I evidence for an association with AD. In a meta-analysis of two population-based and eight case-control studies, RA (57) was found to significantly reduce the risk of AD (OR = 0.60, 95% CI 0.49–0.72, p= 1.06*10^−7^), demonstrating low overall heterogeneity (I^2^ = 33.2%) and no evidence of bias according to Egger's tests. Bae et al.'s Mendelian randomization study (64) further supported a negative correlation between RA and AD by leveraging cross-ethnic genome-wide association study meta-analysis data from participants in Europe and Asia. Additionally, previous research has shown that RA-induced production of granulocyte-macrophage colony-stimulating factor can stimulate the proliferation of monocytes and granulocytes derived from peripheral bone marrow as well as bone marrow-derived microglia (65). Interestingly, these microglia have been observed to reduce amyloid deposition in mouse models of AD (66), suggesting potential long-term protective effects against cognitive impairment. Furthermore, Trzeciak et al. highlighted the similarity in pathological mechanisms between AD and RA regarding immune system activation (67). Therefore, it is plausible that cytokines produced during RA may mediate certain processes while also potentially impacting the blood-brain barrier. Cytokine-mediated amyloid degradation observed in RA could disrupt the bone-joint bond through complex mechanisms that may be linked to Aβ plaques.

The association between diabetes and AD was found to be statistically significant with a low p-value (7.18*10^−18^). A meta-analysis of 20 prospective cohort studies on diabetes reported an increased risk of AD (RR = 1.58, 95% CI 1.43–1.76). Hyperglycemia in diabetic patients leads to the generation of advanced glycation end-products (AGEs) in the brain, which have been identified as important contributors to neurodegeneration (51). AGEs can accelerate amyloid-β aggregation, thereby establishing a link between diabetes and AD (68). Additionally, oxidative stress and neuroinflammation have also been implicated in the development of AD under hyperglycemic conditions (51, 69). Moreover, previous studies have demonstrated that insulin stimulates the release of Aβ into the extracellular space and enhances the expression of insulin-degrading enzyme (IDE), which plays a crucial role in Aβ degradation (70, 71). Interestingly, Michailidis et al. (72) posed a hypothesis that AD, a type 3 diabetes, is a metabolic disease caused by insulin resistance because they are interlinked with the above mechanisms. Not only that, amyloid peptide, which is also called amylin, is produced with β-islet cells and co-secreted with insulin. So, it can be overproduced in T2D and cause insulin resistance in the brain over time, which consequently induce Aβ deposition, Tau phosphorylation, and inflammation (73). However, it is noteworthy that the efficacy of insulin in diabetic patients is relatively limited. Therefore, these aforementioned mechanisms could potentially elucidate the association between diabetes and AD. Implementing lifestyle modifications aimed at preventing individuals at risk of developing diabetes may yield substantial societal benefits by mitigating cognitive decline.

Three factors, namely depression, high homocysteine levels, and low folic acid levels, were classified as Class II evidence for association. The heterogeneity estimate of homocysteine and folic acid levels was not significant (I^2^ < 50%), and the former 95% PI excluded the null value; however, these two factors may have some study bias. High homocysteine levels (Hcy) and low folic acid levels as biomarkers including nine case-control studies that showed a significant association with the development of AD (55). Firstly, a low level of folic acid might impair vitamin B12 intake and degrade cognitive function by aggravating inflammatory reactions (74). Reduced folic acid also damages antioxidant activity in different aspects and may increase homocysteine levels (75). Secondly, homocysteine is an excitatory amino acid that causes cell death by acting on NMDA receptors (76). Homocysteine thiolactone, the metabolite of Hcy, is considered one cause of cellular Hcy toxicity through protein covalent modification inducing protein N-homocysteylation and amyloid formation (77, 78). Li et al. (79) demonstrated that hyperhomocysteinemia epigenetically controls gene expression in the amyloid pathway increasing amyloid deposition in the brain along with phosphorylated tau production (p-Tau), leading to hippocampal neuron death as well as various cognitive deficits. However, more studies are still needed to clarify the relationship between homocysteine/folic acid/vitamin B12 and AD.

The association between depression and risk for AD remained significant, although there may be a bi-directional relationship between them. Studies have suggested that patients with depression may exhibit overperfusion and atrophy in specific brain regions (i.e., anterior cingulate cortex, anterior cuneiform, and parietal lobule) (80), as well as alterations in NMDA receptors (81). These chronic changes could potentially lead to neurodegeneration and the development of AD. Another study (82) demonstrated that depression is accompanied by neuroinflammation, which can activate astrocytes and contribute to amyloid aggregation and p-Tau accumulation. This process is believed to accelerate brain aging and increase the risk of AD (83). On the other hand, several studies (58, 83) have proposed that depression should not only be considered a risk factor for AD but also regarded as a prodromal symptom of AD. It should be given special attention, particularly in older patients. Additionally, due to cognitive decline and decreased self-care abilities later in life, AD can also affect mood and lead to depressive symptoms (84). Therefore, antidepressants may hold potential as a treatment option for AD (85). Further research is needed to fully understand the relationship between depression and AD.

### Strengths and limitations

The strengths of our review lie in the utilization of standardized methods and statistical approaches to conduct an umbrella review, providing a comprehensive summary of current evidence on the association between non-genetic risk factors and AD. This methodology has been previously employed to investigate the credibility of risk factors in relation to other neurodegenerative diseases (86, 87). Additionally, we applied three standard methods (AMSTAR, GRADE, and the evidence classification criteria) to assess the methodological quality, evidence quality, and classification for each risk factor meta-analysis. Upon comparison, inconsistencies were observed between the results derived from GRADE and those obtained using evidence classification criteria. However, it is important to consider that GRADE entails some subjectivity while evidence classification criteria offer a more objective approach; therefore both methods should be taken into account.

However, there are several limitations that need to be acknowledged. Firstly, umbrella reviews require the inclusion of all relevant studies pertaining to the selected topic, which inevitably increases workload and time consumption (17). Secondly, our inclusion criteria encompassed cohort, case-control, or cohort studies that inherently carry a risk of bias. Moreover, it is challenging to address potential issues present in individual systematic reviews such as bias in meta-analysis evaluation due to small sample size or study size (25), which may introduce biases into the evidence classification process. Despite these considerations, we ensured an adequate number of studies were included and the majority of meta-analyses chosen employed NOS for appraising observational studies. Additionally, each meta-analysis was assessed using AMSTAR with most scoring 8 points or higher. In cases where multiple studies discussed the relationship between the same factor and AD, preference was given to meta-analyses with larger sample sizes or more recent studies. Furthermore, statistical methods were extensively utilized for re-evaluating effect sizes under random or fixed-effect models and assessing study bias.

## Conclusion

Based on the evidence classification criteria, we have identified five factors (RA, diabetes, depression, homocysteine levels, and folic acid) that exhibit a robust correlation with the occurrence of AD. These factors can be targeted for improvement through public health policy measures or other interventions. However, it should be noted that while observational evidence has provided convincing or highly suggestive links between modifiable risk factors and AD, translating these findings into tangible benefits for AD patients requires further efforts.

## Electronic supplementary material

Supplementary material, approximately 120 KB.

*Contributors:* YPC and BY designed and supervised the project. SYH selected the articles, extracted and cross-checked the data, contributed to the statistical analysis and wrote the first draft of the manuscript. WMS and SYH selected the articles and extracted and cross-checked the data. XJW, SJL and BC contributed to the statistical analysis. YSH, BY and YPC wrote, revised and discussed the final edition. All authors read and approved the final version of the manuscript.

*Funding:* This study was supported by the National Natural Science Fund of Sichuan (Grant no. 2022NSFSC0749 to B.C.), the 1-3-5 project for disciplines of excellence-Clinical Research Fund, West China Hospital, Sichuan University (Grant No. 2023HXFH032 to Y.P.C.), the Science and Technology Bureau Fund of Sichuan Province (Grant no 2023YFS0269 to Y.P.C.), and the National Innovation and Entrepreneurship Training Program for College Students (Grant No. C2023124417 to X.J.W.).

*Declaration of Competing Interest:* The authors have nothing to disclose.

*Patient consent for publication:* Not required.
