# Supplementary material for: Non-Genetic Risk Factors of Alzheimer's Disease: An Updated Umbrella Review
Source: J Prev Alzheimers Dis. 2024 May 29;11(4):917–27. doi: 10.14283/jpad.2024.100 (PMC11266231; doi:10.14283/jpad.2024.100)
Supplement: Supplementary file 1 — Supplementary material, approximately 120 KB. [file mmc1.docx]

**Supplementary Table 1** Quality assessment (NOS) of 34 articles with 46 risk factors for AD

| **Reference** | **Risk factor** | **Grade** | | |
| --- | --- | --- | --- | --- |
|  |  | High (9) | Moderate (7-8) | Low (<7) |
| Zuin, 2021 | Atrial fibrillation | 2 | 6 | 1 |
| Zhong, 2015 | Ever versus never smoking | 1 | 12 | 9 |
|  | Current versus never smoking | 1 | 9 | 2 |
| Zhao, 2022 | Vitamin E in the diet | 0 | 6 | 3 |
| Yan, 2016 | Pesticide | 0 | 4 | 3 |
| Xie, 2022 | Alcohol consumption | 6 | 6 | 1 |
| Xie, 2020 | Atherosclerosis | 0 | 6 | 4 |
| Wolters, 2018 | CHD | 1 | 4 | 3 |
|  | Heart failure | 1 | 3 | 1 |
| Wang, 2016 | Exposure to aluminum | 1 | 7 | 0 |
| Wang, 2022 | Socioeconomic status | 0 | 5 | 0 |
| Tong, 2022 | ApoA-I | 0 | 15 | 3 |
| Tang, 2019 | Blood lipid levels | 0 | 12 | 15 |
| Su, 2022 | Leisure activities | 1 | 13 | 1 |
| Shi, 2019 | Clusterin | 0 | 6 | 7 |
| Rahmani, 2022 | BMI | 5 | 2 | 1 |
| Qu, H, 2022 | Headache | 0 | 7 | 0 |
| Kim, 2015 | Caffeine | 0 | 4 | 1 |
| Poly, 2020 | Statin | 2 | 17 | 1 |
| Policicchio, 2017 | RA | 2 | 3 | 5 |
| Mehta, 2022 | Depression | 19 | 8 | 0 |
|  | Bone loss | 3 | 0 | 0 |
| Lv, 2018 | BMD | 1 | 1 | 2 |
| Liang, 2021 | Hearing loss | 3 | 2 | 0 |
| Li, 2017 | Head injury | 6 | 20 | 2 |
| Li, 2022 | Albuminuria | 0 | 6 | 0 |
| Kojima, 2016 | Frailty | 0 | 4 | 0 |
| Dun, 2022 | Epilepsy | 2 | 2 | 1 |
| Zhang, 2022 | Cancer | 0 | 9 | 6 |
| Ou, 2020 | HSV-1 | 1 | 4 | 13 |
|  | CMV | 1 | 0 | 5 |
|  | Cpn | 1 | 0 | 10 |
| Kim, 2017 | Peripheral blood BDNF | 0 | 0 | 20 |
| Ou, 2020 | Midlife hypertension | 1 | 2 | 1 |
|  | Late-life hypertension | 2 | 14 | 2 |
|  | AHMs | 2 | 8 | 2 |
| Wang, 2015 | All NSAIDs | 1 | 9 | 6 |
|  | Aspirin | 0 | 6 | 5 |
|  | No-aspirin NSAIDs | 0 | 5 | 3 |
| Li, 2023 | Tooth loss | 2 | 4 | 0 |
| Zhao, 2023 | Antioxidants | 0 | 9 | 3 |
| Yu, 2020 | Stroke | 0 | 4 | 3 |
|  | Vitamin C | 0 | 2 | 4 |
|  | Cognitive activity | 0 | 2 | 4 |
|  | High education | 0 | 1 | 6 |
| Qiao, 2023 | Loneliness | 2 | 1 | 0 |

AD: Alzheimer’s disease, CHD: coronary heart disease, ApoA-I: Napoli protein A1, BMI: body mass index, RA: rheumatoid arthritis, BMD: bone mineral density, HSV-1: herpes simplex virus, CMV: cytomegalovirus, Cpn: chlamydia pneumonia, AHMs: anti-hypertensive medications, NSAIDs: non-steroidal anti-inflammatory drugs

In addition to 34 articles using the Newcastle-Ottawa Scale, 5 articles used 2 tools, 5 articles used other tools for assessment like the Hill Criteria set by the author, the open-access Joanna Briggs Institute criteria tools, and so on, 7 articles can’t provide adequate data about quality assessment and 2 articles didn’t use a tool to some bias.

**Supplement Table 2** Characteristics and quantitative synthesis of 53 eligible articles with 84 meta-analyses for AD

| **Reference** | **Risk factor** | **Number of cases** | **Number of primary studies** | **Effect size** | **Random-effects summary effect size (95% CI)** | **P random** | **95% PI** | **Fixed-effects summary effect size (95% CI)** | **P fixed** |
| --- | --- | --- | --- | --- | --- | --- | --- | --- | --- |
| Zuin, 2021 | Atrial fibrillation | 60785 | 9 | HR | 1.30(1.11-1.47) | 1.00E-04 | 0.87-1.95 | 1.23(1.17-1.27) | 1.84E-30 |
| Zhong, 2015 | Ever versus never smoking | 5816 | 22 | RR | 1.12(0.99-1.26) | 6.50E-02 | 0.56-5.16 | 1.17(1.09-1.24 ) | 2.80E-06 |
|  | Current versus never smoking | 4772 | 12 | OR | 1.41(1.11-1.80) | 5.30E-03 | 0.61-3.23 | 1.34(1.22-1.49) | 1.16E-08 |
| Zhao, 2022 | Vitamin E in the diet | 5409 | 9 | OR | 0.77(0.68-0.87) | 0.000023 | 0.67-0.88 | 0.77(0.68-0.87) | 0.000023 |
| Yan, 2016 | Pesticide | 1050 | 7 | OR | 1.34(1.08-1.67) | 0.0076 | 1.02-1.75 | 1.34(1.08-1.67) | 0.0076 |
| Xie, 2022 | Alcohol consumption | 2321 | 12 | RR | 0.67(0.50-0.92) | 0.0122 | 0.22-2.07 | 0.82(0.77-0.88) | 1.08E-08 |
| Xie, 2020 | Atherosclerosis | 1698 | 10 | OR | 1.50(1.24-1.80) | 1.91E-05 | 0.72-3.13 | 1.13(1.09-1.17) | 1.02E-12 |
| Wolters, 2018 | CHD | >1000* | 8 | RR | 1.09(0.90-1.32) | 0.38 | 0.72-1.65 | 0.93(0.90-0.97) | 0.0001 |
|  | Heart failure | >1000* | 5 | RR | 1.41(0.98-2.03) | 0.61 | 0.52-3.82 | 1.01(0.97-1.05) | 0.0638 |
| Wang, 2016 | Exposure to aluminum | 1383 | 8 | OR | 1.72(1.33-2.21) | 2.79E-05 | 1.19-2.49 | 1.71(1.35-2.18) | 1.05E-05 |
| Wang, 2022 | Socioeconomic status | 7276 | 5 | RR | 1.19(0.70-2.03) | 0.5103 | 0.22-6.53 | 1.16(1.05-1.28) | 0.0027 |
| Tong, 2022 | ApoA-I in plasma | 246 | 5 | OR | 0.12(0.02-0.67) | 0.015 | 0.004-3.21 | 0.52(0.38-0.71) | 4.47E-05 |
|  | ApoA-I in serum | 747 | 9 | OR | 0.12(0.04-0.34) | 6.47E-05 | 0.01-1.5 | 0.11(0.09-0.14) | 8.86E-97 |
|  | ApoA-I in CSF | 201 | 5 | OR | 1.43(0.74-2.76) | 2.70E-01 | 0.48-4.24 | 1.47(1.09-2.00) | 0.012 |
| Tang, 2019 | TC | 2159 | 25 | OR | 1.35(1.02-1.78) | 3.00E-02 | 0.66-2.74 | 1.33(1.20-1.50) | 1.99E-07 |
|  | HDL-C | 1671 | 18 | OR | 0.76(0.54-1.1) | 1.50E-01 | 0.32-1.77 | 0.75(0.66-0.85) | 1.01E-05 |
|  | LDL-C | 1627 | 17 | OR | 1.39(1.03-2.00) | 8.00E-02 | 0.60-3.21 | 1.38(1.22-1.57) | 5.17E-07 |
|  | TG | 1589 | 17 | OR | 1.29(1.12-1.85) | 1.70E-01 | 0.56-2.97 | 1.42(1.25-1.62) | 5.70E-08 |
| Su, 2022 | Leisure activities | 2848 | 15 | RR | 0.82(0.74-0.91) | 0.0001 | 0.55-1.23 | 0.89(0.85-0.93) | 9.16E-07 |
| Shi, 2019 | Clusterin in plasma | 1871 | 13 | OR | 1.41(1.19-2.39) | 0.2 | 0.45-4.4 | 1.69(1.46-1.88) | 3.59E-15 |
|  | Clusterin in CSF | 342 | 3 | OR | 2.7(2.13-3.65) | 8.16E-14 | 0.35-20.37 | 2.79(2.13-3.65) | 8.16E-14 |
| Rahmani, 2022 | Underweight | >1000* | 7 | HR | 1.43(0.86-2.39) | 0.1702 | 0.30-6.75 | 1.48(1.09-2.02) | 0.0121 |
|  | Overweight | >1000* | 8 | HR | 1.02(0.80-1.30) | 0.8724 | 0.46-2.26 | 0.87(0.86-0.88) | 9.75E-121 |
|  | Obese | >1000* | 6 | HR | 1.20(0.76-1.88) | 0.4312 | 0.27-5.23 | 1.37(1.13-1.68) | 0.0015 |
| Qu, 2022 | Headache | >1000* | 7 | OR | 1.53(1.06-2.22) | 0.0248 | 0.52-4.53 | 1.23(1.07-1.40) | 3.20E-03 |
| Kim, 2015 | Caffeine | 590 | 5 | OR | 0.79(0.49-1.27) | 0.3311 | 0.18-3.43 | 0.71(0.57-0.89) | 0.0024 |
| Poly, 2020 | Statin | >1000* | 20 | RR | 0.70(0.60-0.80) | 1.27E-06 | 0.45-2.08 | 0.78(0.72-0.84) | 9.57E-10 |
| Policicchio, 2017 | RA | 1037 | 10 | OR | 0.60(0.49-0.72) | 1.06E-07 | 0.47-0.76 | 0.60(0.49-0.72) | 1.06E-07 |
| Mehta, 2022 | Depression | >1000* | 27 | OR | 1.79(1.46-2.20) | 1.71E-08 | 0.68-4.74 | 1.27(1.25-1.31) | 9.32E-102 |
|  | Bone loss | >1000* | 3 | OR | 1.81(1.28-2.55) | 7.00E-04 | 0.56-5.84 | 1.70(1.35-2.14) | 7.05E-06 |
| Lv, 2018 | BMD | 310 | 4 | OR | 0.11(0.03-0.38) | 5.34E-04 | 0.004-2.45 | 0.09(0.07-0.11) | 4.15E-100 |
| Liang, 2021 | Hearing loss | >1000* | 5 | HR | 2.23(1.33-3.73) | 2.30E-03 | 1.08-4.59 | 2.23(1.33-3.73) | 2.30E-03 |
| Li, 2017 | Head injury | 8166 | 28 | RR | 1.51(1.27-1.80) | 3.24E-06 | 0.63-3.60 | 1.74(1.60-1.89) | 5.41E-39 |
| Li, 2022 | Albuminuria | 741 | 6 | OR | 1.37(1.05-1.79) | 2.04E-02 | 0.77-2.44 | 1.27(1.05-1.54) | 1.48E-02 |
| Kojima, 2016 | Frailty | >1000* | 4 | HR | 1.28(0.88-1.85) | 1.94E-01 | 0.47-3.48 | 1.27(1.00-1.63) | 4.72E-02 |
| Dun, 2022 | Epilepsy | 63261 | 5 | HR | 2.24(1.39-3.59) | 9.00E-04 | 0.59-8.56 | 1.65(1.46-1.87) | 7.80E-16 |
| Zhang, 2022 | Cancer | ＞1000* | 15 | RR | 0.86(0.78-0.94) | 2.20E-03 | 0.61-1.21 | 0.92(0.91-0.93) | 4.27E-36 |
| Ou, 2020 | HSV-1 | 814 | 18 | OR | 1.34(1.02-1.75) | 3.34E-02 | 1.02-1.76 | 1.34(1.02-1.75) | 3.34E-02 |
|  | CMV | 449 | 8 | OR | 1.39(1.05-1.83) | 1.97E-02 | 0.99-1.93 | 1.39(1.05-1.83) | 1.97E-02 |
|  | Cpn | 389 | 11 | OR | 4.56(1.59-13.05) | 4.60E-03 | 0.19-111.21 | 2.77(1.88-4.10) | 3.15E-07 |
| Kim, 2017 | Peripheral blood BDNF | 1455 | 20 | OR | 0.74(0.48-1.15) | 0.18 | 0.98-1.44 | 0.71(0.61-0.83) | 1.15E-05 |
| Ou, 2020 | Midlife hypertension | 2279 | 4 | RR | 1.19(1.08-1.32) | 7.00E-04 | 1.02-1.40 | 1.19(1.08-1.32) | 7.00E-04 |
|  | Late-life hypertension | 4251 | 18 | RR | 0.94(0.85-1.05) | 2.60E-01 | 0.75-1.18 | 0.94(0.87-1.02) | 1.60E-01 |
|  | AHMs | 3424 | 12 | RR | 0.81(0.72-0.91) | 2.00E-04 | 0.59-1.10 | 0.84(0.78-0.90) | 2.23E-07 |
| Wang, 2015 | All NSAIDs | 13407 | 16 | RR | 0.70(0.57-0.85) | 3.00E-04 | 0.34-1.43 | 0.79(0.74-0.84) | 1.19E-12 |
|  | Aspirin | 14303 | 11 | RR | 0.77(0.64-0.93) | 5.80E-03 | 0.45-1.33 | 0.81(0.72-0.90) | 2.00E-04 |
|  | No-aspirin NSAIDs | 29393 | 8 | RR | 0.65(0.48-0.88) | 4.90E-03 | 0.29-1.47 | 0.74(0.62-0.88) | 6.00E-04 |
| Shen, 2015 | High Homocysteine level | 4830 | 9 | RR | 1.89(1.54-2.33) | 1.67E-09 | 1.11-2.81 | 1.89(1.54-2.33) | 1.67E-09 |
|  | Low Folic Acid level | 2070 | 6 | RR | 2.22(1.71-2.89) | 2.73E-09 | 0.95-4.68 | 2.22(1.71-2.89) | 2.73E-09 |
| Du, 2016 | Serum uric acid | 1128 | 20 | OR | 3.86(1.48-10.05) | 6.00E-03 | 0.29-51.22 | 2.08(1.77-2.42) | 1.39E-19 |
| Luo, 2022 | Metformin | >1000* | 10 | OR | 1.15(0.82-1.63) | 4.03E-01 | 0.28-4.70 | 1.11(1.04-1.18) | 1.00E-03 |
| Liu, 2022 | IBD | NR | 5 | RR | 1.65(0.84-3.26) | 1.40E-01 | 0.16-16.62 | 1.70(1.64-1.78) | 6.43E-149 |
| Chai, 2019 | VD deficiency | 14618 | 6 | HR | 1.36(1.13-1.65) | 1.30E-03 | 0.79-2.35 | 1.16(1.08-1.25) | 8.30E-05 |
| Fan, 2019 | Long Sleep | NR | 6 | HR | 1.62(1.19-2.21) | 2.40E-03 | 0.74-3.53 | 1.57(1.34-1.85) | 2.16E-08 |
|  | Short Sleep | NR | 6 | HR | 1.19(0.91-1.56) | 2.10E-01 | 0.58-2.42 | 1.05(0.94-1.10) | 6.90E-01 |
| Li, 2017 | Cu in serum | 2128 | 35 | OR | 3.36(2.03-5.58) | 2.66E-06 | 0.64-17.50 | 2.16(1.92-2.42) | 3.49E-39 |
|  | Zn in serum | 1027 | 22 | OR | 0.62(0.30-1.27) | 1.90E-01 | 0.08-4.37 | 0.62(0.53-0.72) | 1.86E-09 |
|  | Fe in serum | 1379 | 25 | OR | 0.81(0.40-1.67) | 6.00E-01 | 0.10-6.36 | 0.67(0.58-0.78) | 1.15E-07 |
| Lee, 2020 | GA | NR | 17 | OR | 0.92(0.82-1.05) | 2.40E-01 | 0.73-1.16 | 0.92(0.82-1.02) | 1.10E-01 |
| Jalilian, 2018 | Extremely low-frequency magnetic fields | 7362 | 20 | RR | 1.72(1.37-2.15) | 2.10E-06 | 0.69-4.28 | 1.21(1.14-1.28) | 2.89E-09 |
| Dhiman, 2022 | PM_2.5_ | 10633 | 6 | HR | 1.08(0.98-1.18) | 1.00E-01 | 0.82-1.42 | 1.04(1.03-1.05) | 5.62E-22 |
|  | O_3_ | 10165 | 4 | HR | 1.02(0.97-1.07) | 4.50E-01 | 0.84-1.23 | 1.05(1.04-1.05) | 3.15E-161 |
| Qu, 2021 | Plasma/Serum β-carotene levels | 1155 | 14 | OR | 0.96(0.52-1.80) | 9.10E-01 | 0.22-4.14 | 0.66(0.57-0.75) | 8.80E-10 |
|  | Plasma/Serum α-carotene levels | 889 | 10 | OR | 0.69(0.32-1.49) | 3.40E-01 | 0.13-3.63 | 0.89(0.77-1.03) | 1.30E-01 |
|  | Plasma/Serum lycopene | 883 | 10 | OR | 0.80(0.15-4.29) | 8.00E-01 | 0.01-35.49 | 0.72(0.62-0.84) | 2.12E-05 |
|  | Plasma/Serum lutein levels | 846 | 10 | OR | 3.81(1.97-7.39) | 6.89E-05 | 0.92-15.64 | 1.88(1.61-2.21) | 3.50E-15 |
|  | Plasma/Serum β-cryptoxanthin | 860 | 9 | OR | 0.85(0.21-3.46) | 8.20E-01 | 0.73-11.91 | 0.83(0.97-1.38) | 2.00E-02 |
|  | Plasma/Serum zeaxanthin | 846 | 10 | OR | 2.95(1.53-5.59) | 1.00E-03 | 0.16-3.77 | 2.05(1.74-2.39) | 7.16E-19 |
| Zhu, 2021 | DHA | 1560 | 3 | RR | 0.75(0.42-1.34) | 3.40E-01 | 0.08-7.35 | 0.85(0.70-1.03) | 9.00E-02 |
|  | EPA | 1560 | 3 | RR | 0.91(0.66-1.25) | 5.60E-01 | 0.29-2.86 | 0.94(0.81-1.10) | 4.60E-01 |
| Lv, 2016 | Low testosterone | 240 | 7 | RR | 1.49(1.12-1.98) | 6.10E-03 | 0.74-3.00 | 1.36(1.16-1.59) | 1.00E-04 |
| Gudala, 2013 | Diabetes | 4592 | 20 | RR | 1.58(1.43-1.76) | 7.18E-18 | 1.07-1.94 | 1.58(1.43-1.76) | 7.18E-18 |
| Shi, 2018 | Sleep disturbances | 4627 | 9 | RR | 1.70(1.24-2.33) | 1.10E-03 | 0.56-5.16 | 1.26(1.19-1.39) | 1.27E-10 |
| Tsai, 2023 | AMD | 84060 | 6 | HR | 1.21(1.02-1.44) | 3.00E-02 | 0.73-1.99 | 1.23(1.13-1.34) | 1.83E-06 |
| Ahn, 2023 | PPI | 106491 | 5 | RR | 1.15(0.95-1.40) | 1.60E-01 | 0.62-2.13 | 0.98(0.97-1.00) | 0.03 |
| Li, 2023 | Tooth loss | NR | 6 | RR | 1.11(1.03-1.20) | 6.00E-03 | 0.87-1.41 | 1.07(1.04-1.11) | 4.91E-06 |
| Xiong, 2023 | Cataract | >1000 | 9 | HR | 1.17(1.08-1.27) | 7.97E-05 | 0.96-1.43 | 1.09(1.06-1.12) | 3.81E-08 |
| Zhao, 2023 | Antioxidants | 34769 | 12 | RR | 0.83(0.74-0.92) | 7.00E-04 | 0.53-1.31 | 0.85(0.79-0.92) | 4.57E-05 |
| Schliep, 2023 | HDP | >1000 | 3 | HR | 1.40(1.13-1.74) | 1.80E-03 | 0.87-2.25 | 1.40(1.13-1.74) | 1.80E-03 |
| Yu, 2020 | Stroke | 1732 | 7 | RR | 1.38(1.02-1.88) | 3.00E-02 | 0.70-2.74 | 1.46(1.19-1.81) | 4.00E-04 |
|  | Vitamin C | 978 | 6 | RR | 0.84(0.71-1.00) | 4.62E-02 | 0.67-1.06 | 0.84(0.71-1.00) | 4.62E-02 |
|  | Cognitive activity | 532 | 6 | RR | 0.49(0.38-0.63) | 2.36E-08 | 0.27-0.89 | 0.52(0.44-0.62) | 1.79E-13 |
|  | High education | 1727 | 7 | RR | 0.95(0.89-1.00) | 5.70E-02 | 0.79-1.14 | 0.95(0.93-0.98) | 6.41E-05 |
| Qiao, 2022 | Loneliness | 411 | 3 | RR | 1.74(1.30-2.34) | 2.00E-04 | 0.77-3.91 | 1.72(1.32-2.23) | 4.43E-05 |

AD: Alzheimer’s disease, TC: total cholesterol, TG: total triglycerides, HDL-C: high-density lipoprotein, LDL-C: low-density lipoprotein, CHD: coronary heart disease, ApoA-I: Napoli protein A1, BMI: body mass index, RA: rheumatoid arthritis, BMD: bone mineral density, HSV-1: herpes simplex virus, CMV: cytomegalovirus, Cpn: chlamydia pneumonia, AHMs: anti-hypertensive medications, NSAIDs: non-steroidal anti-inflammatory drugs, IBD: inflammatory bowel disease, VD: vitamin D, GA: general anesthesia, PM_2.5_: particulate matter, O_3_: ozone, DHA: docosahexaenoic acid, EPA: eicosapentaenoic acid, AMD: age-related macular degeneration, PPI: proton pump inhibitors, HDP: Hypertensive Disorders of Pregnancy

HR: hazard ratio, OR: odds ratio, RR: relative risk

*For some meta-analyses, authors conducted associations with AD and dementia, We can only roughly judge that the sample size may be greater than 1000.

**Supplementary Table 3** Bias assessment of the 84 meta-analyses of 53 eligible articles

| **Reference** | **Risk factor** | **Effect size** | **Largest study effect size (95% CI)** | **SE** | **I^2^** | **Egger test p-value** | **Observed significant studies** | **Expected significant studies** | **Excess significance test p-value** |
| --- | --- | --- | --- | --- | --- | --- | --- | --- | --- |
| Zuin, 2021 | Atrial fibrillation | HR | 1.12(1.07-1.17) | 0.07 | 85.20% | 0.398 | 8 | 8.12 | NP |
| Zhong, 2015 | Ever versus never smoking | RR | 1.27(1.11-1.45) | 0.06 | 55.90% | 0.005 | 21 | 19.13 | 0.30 |
|  | Current versus never smoking | OR | 1.30(1.11-1.52) | 0.12 | 66.80% | 0.1 | 10 | 8.74 | 0.46 |
| Zhao, 2022 | Vitamin E in the diet | OR | 0.74(0.62-0.88) | 0.06 | 36.80% | 0.348 | 8 | 7.21 | 0.51 |
| Yan, 2016 | Pesticide | OR | 1.42(1.06-1.91) | 0.11 | 0.00% | 0.66 | 3 | 4.46 | NP |
| Xie, 2022 | Alcohol consumption | RR | 1.10(0.99-1.22) | 0.16 | 88.30% | 0.16 | 10 | 10.57 | NP |
| Xie, 2020 | Atherosclerosis | OR | 1.06(1.04-2.46) | 0.09 | 88.20% | 0.004 | 10 | 8.9 | 0.27 |
| Wolters, 2018 | CHD | RR | 0.92(0.89-0.96) | 0.09 | 30.20% | 0.04 | 6 | 5.1 | 0.50 |
|  | Heart failure | RR | 1.00(0.96-1.04) | 0.19 | 73.80% | 0.03 | 4 | 2.54 | 0.19 |
| Wang, Z, 2016 | Exposure to aluminum | OR | 1.70(1.15-2.50) | 0.13 | 6.20% | 0.81 | 5 | 3.87 | 0.42 |
| Wang, 2022 | Socioeconomic status | RR | 1.18(1.06-1.31) | 0.27 | 83.30% | 0.04 | 5 | 3.77 | 0.20 |
| Tong, 2022 | ApoA-I in plasma | OR | 1.00(0.68-1.47) | 0.46 | 94.40% | 0.069 | 4 | 1.86 | 0.06 |
|  | ApoA-I in serum | OR | 0.75(0.46-1.22) | 0.29 | 95.90% | 0.96 | 9 | 13.84 | NP |
|  | ApoA-I in CSF | OR | 1.49(1.01-2.19) | 0.18 | 65.30% | 0.901 | 2 | 1.04 | 0.30 |
| Tang, Q, 2019 | TC | OR | 1.31(1.04-1.78) | 0.08 | 82% | 0.875 | 10 | 4.49 | 0.004 |
|  | HDL-C | OR | 0.68(0.49-0.95) | 0.1 | 87% | 0.926 | 8 | 3.24 | 0.004 |
|  | LDL-C | OR | 1.16(0.83-1.62) | 0.1 | 87% | 0.887 | 4 | 3.86 | 0.94 |
|  | TG | OR | 1.34(1.04-1.87) | 0.1 | 87% | 0.454 | 8 | 4.34 | 0.04 |
| Su, 2022 | Leisure activities | RR | 0.98(0.89-1.08) | 0.05 | 69.70% | 0.013 | 19 | 18.66 | 0.56 |
| Shi, X, 2019 | Clusterin in plasma | OR | 1.01(1.28-1.32) | 0.15 | 93% | 0.435 | 6 | 6.41 | NP |
|  | Clusterin in CSF | OR | 2.86(2.16-3.8) | 0.47 | 0% | NE | 1 | 1.66 | NP |
| Rahmani, 2022 | Underweight | HR | 2.3(1.17-4.84) | 0.26 | 60.40% | 0.52 | 4 | 2.89 | 0.42 |
|  | Overweight | HR | 0.87(0.86-0.88) | 0.12 | 87.40% | 0.34 | 9 | 8.48 | 0.65 |
|  | Obese | HR | 3.10(2.19-4.38) | 0.23 | 81.90% | 0.47 | 5 | 4.52 | 0.74 |
| Qu, H, 2022 | Headache | OR | 1.07(0.89-1.28) | 0.19 | 70.40% | 0.087 | 4 | 4.2 | NP |
| Kim, 2015 | Caffeine | OR | 0.69(0.50-0.96) | 0.25 | 71.00% | 0.63 | 4 | 3.17 | 0.44 |
| Poly, 2020 | Statin | RR | 0.85(0.76-0.95) | 0.07 | 47.90% | <0.002 | 15 | 17.45 | NP |
| Policicchio, 2017 | RA | OR | 0.61(0.43-0.87) | 0.09 | 33.20% | 0.668 | 6 | 5.87 | 0.93 |
| Mehta, 2022 | Depression | OR | 1.39(1.35-1.44) | 0.1 | 94.50% | 0.06 | 21 | 15.47 | 0.04 |
|  | Bone loss | OR | 1.47(1.10-1.97) | 0.18 | 43.80% | NE | 3 | 2.38 | 0.38 |
| Lv, 2018 | BMD | OR | 0.02(0.01-0.03) | 0.36 | 95.90% | NE | 6 | 6.85 | NP |
| Liang, 2021 | Hearing loss | HR | 1.85(0.93-3.69) | 0.26 | 1.60% | 0.52 | 1 | 1.29 | NP |
| Li, 2017 | Head injury | RR | 2.70(2.20-3.31) | 0.08 | 70.10% | 0.139 | 22 | 23.74 | NP |
| Li, 2022 | Albuminuria | RR | 1.01(0.77-1.32) | 0.13 | 17.40% | 0.011 | 4 | 3.52 | 0.69 |
| Kojima, 2016 | Frailty | HR | 1.08(0.74-1.57) | 0.18 | 51.10% | 0.76 | 3 | 3.25 | NP |
| Dun, 2022 | Epilepsy | HR | 1.50(1.31-1.72) | 0.24 | 78.10% | 0.95 | 3 | 2.55 | 0.68 |
| Zhang, 2022 | Cancer | RR | 0.94(0.92-0.96) | 0.04 | 91.90% | 0.31 | 14 | 15.04 | NP |
| Ou, 2020 | HSV-1 | OR | 1.37(0.69-2.73) | 0.13 | 0.00% | 0.99 | 5 | 1.89 | 0.02 |
|  | CMV | OR | 1.46(1.01-2.11) | 0.14 | 12.70% | 0.13 | 2 | 3.47 | NP |
|  | Cpn | OR | 2.44(1.23-4.85) | 0.54 | 71.40% | 0.346 | 3 | 1.56 | 0.21 |
| Kim, 2017 | Peripheral blood BDNF | OR | 1.11(0.78-1.58) | 0.12 | 85.80% | 0.776 | 14 | 3.29 | <0.001 |
| Ou, 2020 | Midlife hypertension | RR | 1.17(1.05-1.30) | 0.05 | 0.00% | 0.29 | 4 | 3.17 | 0.31 |
|  | Late-life hypertension | RR | 0.96(0.84-1.09) | 0.05 | 24.70% | 0.003 | 18 | 14.83 | 0.05 |
|  | AHMs | RR | 1.00(0.88-1.13) | 0.06 | 52.10% | 0.97 | 12 | 11.74 | 0.60 |
| Wang, 2015 | All NSAIDs | RR | 0.76(0.68-0.85) | 0.09 | 75.00% | 0.099 | 14 | 14.88 | NP |
|  | Aspirin | RR | 0.74(0.57-0.97) | 0.09 | 52.10% | 0.66 | 10 | 10.84 | NP |
|  | No-aspirin NSAIDs | RR | 1.19(0.87-1.62) | 0.15 | 63.40% | 0.62 | 6 | 7.01 | NP |
| Shen, 2015 | High homocysteine level | RR | 1.8(1.30-2.50) | 0.1 | 0.00% | 0.005 | 5 | 4.54 | 0.76 |
|  | Low folic acid level | RR | 2.30(1.40-3.79) | 0.13 | 0.00% | 0.24 | 5 | 4.05 | 0.41 |
| Kunutsor, 2022 | Serum uric acid | OR | 1.65(1.21-2.25) | 0.27 | 97.10% | 0.215 | 16 | 8.97 | 0.002 |
| Luo, 2022 | Metformin | OR | 0.85(0.76-0.95) | 0.17 | 94.20% | 0.752 | 9 | 8.35 | 0.58 |
| Liu, 2022 | IBD | RR | 2.46(2.33-2.59) | 0.34 | 99.20% | 0.01 | 5 | 4.09 | 0.29 |
| Chai, 2019 | VD deficiency | HR | 1.10(1.01-1.20) | 0.09 | 53.20% | 0.076 | 8 | 6.04 | 0.16 |
| Fan, 2019 | Long Sleep | HR | 1.54(1.28-1.86) | 0.16 | 44.40% | 0.76 | 4 | 3.63 | 0.76 |
|  | Short Sleep | HR | 0.99(0.91-1.07) | 0.14 | 57.80% | 0.76 | 5 | 3.97 | 0.37 |
| Li, 2017 | Cu in serum | OR | 3.12(2.37-4.11) | 0.14 | 94.40% | 0.077 | 22 | 16.88 | 0.08 |
|  | Zn in serum | OR | 0.67(0.51-0.89) | 0.2 | 94.70% | 0.992 | 14 | 4.96 | <0.001 |
|  | Fe in serum | OR | 0.50(0.30-0.82) | 0.2 | 95.70% | 0.643 | 11 | 4.64 | 0.001 |
| Lee, 2020 | GA | OR | 0.88(0.70-1.10) | 0.06 | 20.90% | 0.72 | 12 | 11.51 | 0.80 |
| Jalilian, 2018 | Extremely low-frequency magnetic fields | RR | 1.12(1.04-1.21) | 0.11 | 61.00% | 0.33 | 13 | 10.57 | 0.36 |
| Dhiman, 2022 | PM_2.5_ | HR | 0.98(0.97-0.99) | 0.05 | 99.20% | 0.34 | 5 | 4.69 | 0.56 |
|  | O_3_ | HR | 1.09(1.08-1.10) | 0.03 | 99.50% | 0.34 | 4 | 4 | NP |
| Qu, 2021 | Plasma/Serum β-carotene levels | OR | 0.82(0.62-1.09) | 0.18 | 94.40% | 0.38 | 6 | 4.81 | 0.52 |
|  | Plasma/Serum α-carotene levels | OR | 1.12(0.84-1.48) | 0.22 | 95.50% | 0.639 | 7 | 0.86 | <0.001 |
|  | Plasma/Serum lycopene | OR | 0.83(0.63-1.11) | 0.47 | 99.10% | 0.74 | 8 | 2.7 | <0.001 |
|  | Plasma/Serum lutein levels | OR | 1.13(0.85-1.49) | 0.19 | 92.10% | 0.067 | 7 | 5.08 | 0.27 |
|  | Plasma/Serum β-cryptoxanthin | OR | 0.86(0.64-1.13) | 0.39 | 98.70% | 0.81 | 5 | 1.18 | <0.001 |
|  | Plasma/Serum zeaxanthin | OR | 1.24(1.06-1.64) | 0.18 | 92.00% | 0.31 | 5 | 5.6 | NP |
| Zhu, 2021 | DHA | RR | 0.73(0.57-0.94) | 0.3 | 76.20% | NE | 2 | 2.93 | NP |
|  | EPA | RR | 1.10(0.90-1.35) | 0.16 | 64.80% | NE | 2 | 2.47 | NP |
| Lv, 2016 | Low testosterone | RR | 1.27(1.03-1.57) | 0.15 | 47.20% | 0.15 | 4 | 3.92 | 0.95 |
| Gudala, 2013 | Diabetes | RR | 1.60(1.29-1.98) | 0.05 | 13.30% | 0.93 | 16 | 16.14 | NP |
| Shi, 2018 | Sleep disturbances | RR | 1.19(1.06-1.34) | 0.16 | 75.70% | 0.93 | 9 | 6.9 | 0.19 |
| Tsai, 2023 | AMD | HR | 1.23(1.04-1.46) | 0.08 | 69.30% | 0.015 | 6 | 6 | NP |
| Ahn, 2023 | PPI | RR | 1.02(1.00-1.04) | 0.09 | 96.90% | 0.38 | 5 | 4.96 | 0.85 |
| Li, 2023 | Tooth loss | RR | 1.00(0.95-1.06) | 0.04 | 61.40% | 0.003 | 11 | 9.42 | 0.27 |
| Xiong, 2023 | Cataract | HR | 1.06(1.03-1.10) | 0.04 | 48.80% | 0.6 | 10 | 9.97 | 0.86 |
| Zhao, 2023 | Antioxidants | RR | 0.75(0.57-1.00) | 0.06 | 45.70% | <0.0001 | 29 | 27.42 | 0.43 |
| Schliep, 2023 | HDP | HR | 1.30(0.97-1.74) | 0.11 | 14.00% | NE | 2 | 2.08 | NP |
| Yu, 2020 | Stroke | RR | 1.79(1.28-2.50) | 0.15 | 28.00% | 0.98 | 4 | 4.39 | NP |
|  | Vitamin C | RR | 0.95(0.72-1.25) | 0.09 | 0.00% | 0.51 | 6 | 5.57 | 0.5 |
|  | Cognitive activity | RR | 0.58(0.44-0.77) | 0.12 | 35.80% | 0.38 | 5 | 5.57 | NP |
|  | High education | RR | 0.94(0.90-0.98) | 0.03 | 74.20% | 0.5 | 7 | 7 | NP |
| Qiao, 2022 | Loneliness | RR | 1.51(1.07-2.13) | 0.15 | 17.80% | NE | 3 | 2.48 | 0.43 |

TC: total cholesterol, TG: total triglycerides, HDL-C: high-density lipoprotein, LDL-C: low-density lipoprotein, CHD: coronary heart disease,

ApoA-I: Napoli protein A1, BMI: body mass index, RA: rheumatoid arthritis, BMD: bone mineral density, HSV-1: herpes simplex virus, CMV: cytomegalovirus, Cpn: chlamydia pneumonia, AHMs: anti-hypertensive medications, NSAIDs: non-steroidal anti-inflammatory drugs, IBD: inflammatory bowel disease, VD: vitamin D, GA: general anesthesia, PM_2.5_: particulate matter, O_3_: ozone, DHA: docosahexaenoic acid, EPA: eicosapentaenoic acid, AMD: age-related macular degeneration, PPI: proton pump inhibitors, HDP: Hypertensive Disorders of Pregnancy

HR: hazard ratio, OR: odds ratio, RR: relative risk

NE: not estimable because less than three studies were available, NP: not pertinent because the number of expected significant studies was larger than the number of observed significant studies

**Supplementary Table 4** Assessment of AMSTAR scores

| Reference | Risk factor | A priori design provided | Duplicate study selection and data extraction | At least two electronic databases searched | Status of publication used as an inclusion criterion | List of included and excluded studies provided | Characteristics of included studies provided | Scientific quality of included studies assessed | Scientific quality of the included studies used appropriately to form conclusions | Appropriate methods to combine studies | Publication bias assessed | Conflict of interest included | Total AMSTAR Score |
| --- | --- | --- | --- | --- | --- | --- | --- | --- | --- | --- | --- | --- | --- |
| Zuin, 2021 | atrial fibrillation | 0 | 1 | 1 | 0 | 1 | 1 | 1 | 0 | 1 | 1 | 1 | 8 |
| Zhong, 2015 | ever versus never somking | 0 | 1 | 1 | 0 | 1 | 1 | 1 | 1 | 1 | 1 | 1 | 9 |
|  | current versus never smoking | 0 | 1 | 1 | 0 | 1 | 1 | 1 | 1 | 1 | 1 | 1 | 9 |
| Zhao, 2022 | vitamin E in diet | 0 | 1 | 1 | 0 | 1 | 1 | 1 | 1 | 1 | 1 | 1 | 9 |
| Yan, 2016 | pesticide | 1 | 1 | 1 | 0 | 1 | 1 | 1 | 1 | 1 | 1 | 1 | 10 |
| Xie, 2022 | alcohol consumption | 0 | 1 | 1 | 0 | 1 | 1 | 1 | 1 | 1 | 0 | 1 | 8 |
| Xie, 2020 | atherosclerosis | 0 | 1 | 1 | 0 | 1 | 1 | 1 | 0 | 1 | 1 | 1 | 8 |
| Wolters, 2018 | CHD | 0 | 1 | 1 | 0 | 1 | 1 | 1 | 1 | 1 | 1 | 1 | 9 |
|  | heart failure | 0 | 1 | 1 | 0 | 1 | 1 | 1 | 1 | 1 | 1 | 1 | 9 |
| Wang, 2016 | exposure to aluminum | 0 | 1 | 1 | 0 | 1 | 1 | 1 | 0 | 1 | 1 | 1 | 8 |
| Wang, 2022 | socioeconomic status | 1 | 1 | 1 | 0 | 1 | 1 | 1 | 1 | 1 | 1 | 1 | 10 |
| Tong, 2022 | ApoA-I in plasma | 1 | 1 | 1 | 0 | 1 | 1 | 1 | 1 | 1 | 1 | 1 | 10 |
|  | ApoA-I in serum | 1 | 1 | 1 | 0 | 1 | 1 | 1 | 1 | 1 | 1 | 1 | 10 |
|  | ApoA-I in CSF | 1 | 1 | 1 | 0 | 1 | 1 | 1 | 1 | 1 | 1 | 1 | 10 |
| Tang, Q, 2019 | TC | 0 | 1 | 1 | 1 | 1 | 1 | 1 | 0 | 1 | 1 | 1 | 9 |
|  | HDL-C | 0 | 1 | 1 | 1 | 1 | 1 | 1 | 0 | 1 | 1 | 1 | 9 |
|  | LDL-C | 0 | 1 | 1 | 1 | 1 | 1 | 1 | 0 | 1 | 1 | 1 | 9 |
|  | TG | 0 | 1 | 1 | 1 | 1 | 1 | 1 | 0 | 1 | 1 | 1 | 9 |
| Su, 2022 | leisure activities | 1 | 1 | 1 | 0 | 1 | 1 | 1 | 0 | 1 | 1 | 1 | 9 |
| Shi, X, 2019 | clusterin in plasma | 0 | 0 | 1 | 0 | 1 | 1 | 1 | 1 | 1 | 1 | 1 | 8 |
|  | clusterin in CSF | 0 | 0 | 1 | 0 | 1 | 1 | 1 | 1 | 1 | 1 | 1 | 8 |
| Rahmani, 2022 | underweight | 0 | 1 | 1 | 0 | 1 | 1 | 0 | 1 | 1 | 1 | 1 | 8 |
|  | overweight | 0 | 1 | 1 | 0 | 1 | 1 | 0 | 1 | 1 | 1 | 1 | 8 |
|  | obese | 0 | 1 | 1 | 0 | 1 | 1 | 0 | 1 | 1 | 1 | 1 | 8 |
| Qu, H, 2022 | headache | 1 | 1 | 1 | 0 | 1 | 1 | 1 | 0 | 1 | 1 | 1 | 9 |
| Kim, 2015 | caffeine | 0 | 1 | 1 | 0 | 1 | 1 | 1 | 1 | 1 | 1 | 0 | 8 |
| Poly, 2020 | statin | 0 | 1 | 1 | 0 | 1 | 1 | 1 | 1 | 1 | 1 | 0 | 8 |
| Policicchio, 2017 | RA | 0 | 0 | 1 | 1 | 1 | 1 | 0 | 1 | 1 | 1 | 1 | 8 |
| Mehta, 2022 | depression | 1 | 1 | 1 | 1 | 1 | 1 | 1 | 1 | 1 | 1 | 1 | 11 |
|  | bone loss | 1 | 1 | 1 | 1 | 1 | 1 | 1 | 1 | 1 | 0 | 1 | 10 |
| Lv, 2018 | BMD | 0 | 1 | 1 | 1 | 1 | 1 | 0 | 0 | 1 | 1 | 1 | 8 |
| Liang, 2021 | hearing loss | 0 | 1 | 1 | 0 | 1 | 1 | 1 | 0 | 1 | 1 | 1 | 8 |
| Li, 2017 | head injury | 0 | 0 | 1 | 0 | 1 | 1 | 0 | 0 | 1 | 1 | 0 | 5 |
| Li, 2022 | albuminuria | 0 | 1 | 1 | 0 | 1 | 1 | 1 | 0 | 1 | 1 | 1 | 8 |
| Kojima, 2016 | frailty | 0 | 1 | 1 | 0 | 1 | 1 | 1 | 0 | 1 | 1 | 0 | 7 |
| Dun, 2022 | epilepsy | 1 | 1 | 1 | 0 | 1 | 1 | 1 | 0 | 1 | 1 | 1 | 9 |
| Zhang, 2022 | cancer | 1 | 1 | 1 | 0 | 1 | 1 | 1 | 0 | 1 | 1 | 1 | 9 |
| Ou, 2020 | HSV-1 | 0 | 1 | 1 | 0 | 1 | 1 | 1 | 0 | 1 | 1 | 1 | 8 |
|  | CMV | 0 | 1 | 1 | 0 | 1 | 1 | 1 | 0 | 1 | 0 | 1 | 7 |
|  | Cpn | 0 | 1 | 1 | 0 | 1 | 1 | 1 | 0 | 1 | 1 | 1 | 8 |
| Kim, 2017 | peripheral blood BDNF | 0 | 1 | 1 | 0 | 1 | 1 | 1 | 0 | 1 | 1 | 1 | 8 |
| Ou, 2020 | midlife hypertension | 0 | 1 | 1 | 0 | 1 | 1 | 1 | 1 | 1 | 0 | 1 | 8 |
|  | late-life hypertension | 0 | 1 | 1 | 0 | 1 | 1 | 1 | 1 | 1 | 1 | 1 | 9 |
|  | AHMs | 0 | 1 | 1 | 0 | 1 | 1 | 1 | 1 | 1 | 1 | 1 | 9 |
| Wang, 2015 | all NSAIDs | 0 | 1 | 1 | 1 | 1 | 1 | 0 | 1 | 1 | 1 | 1 | 9 |
|  | aspirin | 0 | 1 | 1 | 1 | 1 | 1 | 0 | 1 | 1 | 0 | 1 | 8 |
|  | no-aspirin NSAIDs | 0 | 1 | 1 | 1 | 1 | 1 | 0 | 1 | 1 | 0 | 1 | 8 |
| Shen, 2015 | Homocysteine | 0 | 0 | 1 | 1 | 1 | 1 | 0 | 0 | 1 | 0 | 1 | 6 |
|  | Folic Acid | 0 | 0 | 1 | 1 | 1 | 1 | 0 | 0 | 1 | 0 | 1 | 6 |
| Kunutsor, 2022 | serum uric acid | 0 | 1 | 1 | 0 | 1 | 1 | 1 | 0 | 1 | 1 | 1 | 8 |
| Luo, 2022 | Metformin | 0 | 1 | 1 | 0 | 1 | 1 | 1 | 1 | 1 | 1 | 1 | 9 |
| Liu, 2022 | IBD | 1 | 1 | 1 | 0 | 1 | 1 | 1 | 0 | 1 | 0 | 1 | 8 |
| Chai, 2019 | VD | 0 | 1 | 1 | 1 | 1 | 1 | 1 | 1 | 1 | 1 | 1 | 10 |
| Fan, 2019 | Long Sleep | 0 | 1 | 1 | 1 | 1 | 1 | 1 | 1 | 1 | 1 | 0 | 9 |
|  | Short Sleep | 0 | 1 | 1 | 1 | 1 | 1 | 1 | 1 | 1 | 1 | 0 | 9 |
| Li, 2017 | Cu in serum | 0 | 1 | 1 | 1 | 1 | 1 | 1 | 1 | 1 | 1 | 1 | 10 |
|  | Zn in serum | 0 | 1 | 1 | 1 | 1 | 1 | 1 | 1 | 1 | 1 | 1 | 10 |
|  | Fe in serum | 0 | 1 | 1 | 1 | 1 | 1 | 1 | 1 | 1 | 1 | 1 | 10 |
| Lee, 2020 | GA | 1 | 0 | 1 | 0 | 1 | 1 | 1 | 0 | 1 | 1 | 1 | 8 |
| Jalilian, 2018 | extremely low frequency magnetic fields | 0 | 1 | 1 | 1 | 1 | 1 | 0 | 1 | 1 | 1 | 1 | 9 |
| Dhiman, 2022 | PM2.5 | 1 | 1 | 1 | 1 | 1 | 1 | 0 | 0 | 1 | 1 | 1 | 9 |
|  | O3 | 1 | 1 | 1 | 1 | 1 | 1 | 0 | 0 | 1 | 1 | 1 | 9 |
| Qu, 2021 | plasma/serum β-carotene levels | 0 | 1 | 1 | 1 | 1 | 1 | 0 | 1 | 1 | 1 | 1 | 9 |
|  | plasma/serum α-carotene levels | 0 | 1 | 1 | 1 | 1 | 1 | 0 | 1 | 1 | 1 | 1 | 9 |
|  | plasma/serum lycopene | 0 | 1 | 1 | 1 | 1 | 1 | 0 | 1 | 1 | 1 | 1 | 9 |
|  | plasma/serum lutein levels | 0 | 1 | 1 | 1 | 1 | 1 | 0 | 1 | 1 | 1 | 1 | 9 |
|  | plasma/serum β-cryptoxanthin | 0 | 1 | 1 | 1 | 1 | 1 | 0 | 1 | 1 | 1 | 1 | 9 |
|  | plasma/serum zeaxanthin | 0 | 1 | 1 | 1 | 1 | 1 | 0 | 1 | 1 | 1 | 1 | 9 |
| Zhu, 2021 | DHA | 0 | 1 | 1 | 0 | 1 | 1 | 1 | 0 | 1 | 0 | 1 | 7 |
|  | EPA | 0 | 1 | 1 | 0 | 1 | 1 | 1 | 0 | 1 | 0 | 1 | 7 |
| Lv, 2016 | low testosterone | 0 | 1 | 1 | 0 | 1 | 1 | 1 | 1 | 1 | 1 | 1 | 9 |
| Gudala, 2013 | diabetes | 0 | 1 | 1 | 1 | 1 | 1 | 1 | 1 | 1 | 1 | 1 | 10 |
| Shi, 2018 | sleep disturbances | 0 | 1 | 1 | 0 | 1 | 1 | 0 | 1 | 1 | 1 | 1 | 8 |
| Tsai, 2023 | AMD | 1 | 1 | 1 | 0 | 1 | 1 | 1 | 0 | 1 | 0 | 1 | 8 |
| Ahn, 2023 | PPI | 1 | 1 | 1 | 0 | 1 | 1 | 1 | 0 | 1 | 1 | 1 | 9 |
| Li, 2023 | Tooth loss | 0 | 1 | 1 | 0 | 1 | 1 | 1 | 0 | 1 | 0 | 1 | 7 |
| Xiong, 2023 | Cataract | 1 | 1 | 1 | 0 | 1 | 1 | 1 | 1 | 1 | 1 | 1 | 10 |
| Zhao, 2023 | Antioxidants | 0 | 1 | 1 | 1 | 1 | 1 | 1 | 0 | 1 | 1 | 1 | 9 |
| Schliep, 2023 | HDP | 1 | 1 | 1 | 1 | 1 | 1 | 1 | 0 | 1 | 0 | 1 | 9 |
| Yu, 2019 | Stroke | 0 | 1 | 1 | 1 | 1 | 1 | 1 | 1 | 1 | 0 | 1 | 9 |
|  | Vitamin C | 0 | 1 | 1 | 1 | 1 | 1 | 1 | 1 | 1 | 1 | 1 | 10 |
|  | Cognitive activity | 0 | 1 | 1 | 1 | 1 | 1 | 1 | 1 | 1 | 0 | 1 | 9 |
|  | High education | 0 | 1 | 1 | 1 | 1 | 1 | 1 | 1 | 1 | 0 | 1 | 9 |
| Qiao, 2022 | Loneliness | 0 | 1 | 1 | 0 | 1 | 1 | 1 | 0 | 1 | 1 | 1 | 8 |

TC: total cholesterol, TG: total triglycerides, HDL-C: high-density lipoprotein, LDL-C: low-density lipoprotein, CHD: coronary heart disease, ApoA-I: Napoli protein A1, BMI: body mass index, RA: rheumatoid arthritis, BMD: bone mineral density, HSV-1: herpes simplex virus, CMV: cytomegalovirus, Cpn: chlamydia pneumonia, AHMs: anti-hypertensive medications, NSAIDs: non-steroidal anti-inflammatory drugs, IBD: inflammatory bowel disease, VD: vitamin D, GA: general anesthesia, PM_2.5_: particulate matter, O_3_: ozone, DHA: docosahexaenoic acid, EPA: eicosapentaenoic acid, AMD: age-related macular degeneration, PPI: proton pump inhibitors, HDP: Hypertensive Disorders of Pregnancy

**Supplementary Table 5** Assessment of GRADE

| **Reference** | **Risk factor** | **No. of studies** | **RCT** | **Cohort** | **Case-control/cross-sectional** | **Risk of bias** | **Inconsistency** | **Indirectness** | **Imprecision** | **Publication bias** | **Plausible confounding** | **Magnitude of effect** | **Dose-response gradient** | **Quality** |
| --- | --- | --- | --- | --- | --- | --- | --- | --- | --- | --- | --- | --- | --- | --- |
| Zuin, 2021 | atrial fibrillation | 9 | 0 | 9 | 0 | No serious | No serious | No serious | No serious | No | Not reduce effect | No | No | Low |
| Zhong, 2015 | ever versus never somking | 22 | 0 | 22 | 0 | Serious | No serious | Serious | No serious | No | Not reduce effect | No | No | Very low |
|  | current versus never smoking | 12 | 0 | 12 | 0 | Serious | No serious | Serious | No serious | No | Not reduce effect | No | No | Very low |
| Zhao, 2022 | vitamin E in diet | 9 | 0 | 8 | 1 | Serious | No serious | No serious | No serious | No | Not reduce effect | No | No | Very low |
| Yan, 2016 | pesticide | 7 | 0 | 3 | 4 | Serious | No serious | No serious | No serious | No | Not reduce effect | No | No | Very low |
| Xie, 2022 | alcohol consumption | 12 | 0 | 8 | 4 | Serious | No serious | Serious | No serious | No | Not reduce effect | No | No | Very low |
| Xie, 2020 | atherosclerosis | 10 | 0 | 3 | 7 | No serious | No serious | No serious | No serious | Strongly suspected | Not reduce effect | No | No | Very low |
| Wolters, 2018 | CHD | 8 | 0 | 8 | 0 | No serious | No serious | No serious | No serious | No | Not reduce effect | No | No | Low |
|  | heart failure | 5 | 0 | 5 | 0 | No serious | Serious | No serious | No serious | Strongly suspected | Not reduce effect | No | No | Very low |
| Wang, Z, 2016 | exposure to aluminum | 8 | 0 | 8 | 0 | No serious | No serious | No serious | No serious | No | Not reduce effect | No | No | Low |
| Wang, 2022 | socioeconomic status | 5 | 0 | 5 | 0 | No serious | No serious | No serious | Serious | No | Not reduce effect | No | No | Very low |
| Tong, 2022 | ApoA-I in plasma | 5 | 0 | 0 | 5 | No serious | No serious | No serious | Serious | No | Not reduce effect | No | No | Very low |
|  | ApoA-I in serum | 9 | 0 | 0 | 9 | No serious | No serious | No serious | Serious | No | Not reduce effect | No | No | Very low |
|  | ApoA-I in CSF | 5 | 0 | 0 | 5 | No serious | No serious | No serious | Serious | No | Not reduce effect | No | No | Very low |
| Tang, Q, 2019 | TC | 25 | 0 | 0 | 25 | No serious | Serious | No serious | No serious | No | Not reduce effect | No | No | Very low |
|  | HDL-C | 18 | 0 | 0 | 18 | No serious | Serious | Serious | No serious | No | Not reduce effect | No | No | Very low |
|  | LDL-C | 17 | 0 | 0 | 17 | No serious | Serious | Serious | No serious | No | Not reduce effect | No | No | Very low |
|  | TG | 17 | 0 | 0 | 17 | No serious | Serious | No serious | No serious | No | Not reduce effect | No | No | Very low |
| Su, 2022 | leisure activities | 15 | 0 | 15 | 0 | No serious | No serious | No serious | No serious | No | Not reduce effect | No | No | Low |
| Shi, X, 2019 | clusterin in plasma | 13 | 0 | 3 | 10 | No serious | Serious | No serious | No serious | No | Not reduce effect | No | No | Very low |
|  | clusterin in CSF | 3 | 0 | 0 | 3 | No serious | Serious | No serious | Serious | NA | Not reduce effect | No | No | Very low |
| Rahmani, 2022 | underweight | 7 | 0 | 7 | 0 | No serious | Serious | No serious | Serious | No | Not reduce effect | No | No | Very low |
|  | overweight | 8 | 0 | 8 | 0 | No serious | Serious | No serious | No serious | No | Not reduce effect | No | No | Very low |
|  | obese | 6 | 0 | 6 | 0 | No serious | Serious | No serious | Serious | No | Not reduce effect | No | No | Very low |
| Qu, H, 2022 | headache | 7 | 0 | 7 | 0 | Serious | No serious | No serious | No serious | No | Not reduce effect | No | No | Very low |
| Kim, 2015 | caffeine | 5 | 0 | 2 | 3 | Serious | No serious | No serious | Serious | No | Not reduce effect | No | No | Very low |
| Poly, 2020 | statin | 20 | 0 | 15 | 5 | No serious | No serious | No serious | No serious | No | Not reduce effect | No | No | Low |
| Policicchio, 2017 | RA | 10 | 0 | 2 | 8 | No serious | No serious | No serious | No serious | No | Not reduce effect | No | No | Low |
| Mehta, 2022 | depression | 27 | 0 | 27 | 0 | No serious | No serious | No serious | No serious | No | Not reduce effect | No | No | Low |
|  | bone loss | 3 | 0 | 3 | 0 | No serious | Serious | No serious | Serious | NA | Not reduce effect | No | No | Very low |
| Lv, 2018 | BMD | 4 | 0 | 2 | 2 | Serious | No serious | No serious | Serious | No | Not reduce effect | No | No | Very low |
| Liang, 2021 | hearing loss | 5 | 0 | 5 | 0 | No serious | No serious | No serious | No serious | No | Not reduce effect | No | No | Low |
| Li, 2017 | head injury | 28 | 0 | 12 | 16 | Serious | No serious | No serious | No serious | No | Not reduce effect | No | No | Very low |
| Li, 2022 | albuminuria | 6 | 0 | 6 | 0 | No serious | No serious | No serious | Serious | Strongly suspected | Not reduce effect | No | No | Very low |
| Kojima, 2016 | frailty | 4 | 0 | 4 | 0 | No serious | No serious | No serious | No serious | No | Not reduce effect | No | No | Low |
| Dun, 2022 | epilepsy | 5 | 0 | 5 | 0 | No serious | No serious | No serious | No serious | No | Not reduce effect | No | No | Low |
| Zhang, 2022 | cancer | 15 | 0 | 15 | 0 | No serious | Serious | No serious | No serious | No | Not reduce effect | No | No | Very low |
| Ou, 2020 | HSV-1 | 18 | 0 | 0 | 18 | Serious | No serious | No serious | Serious | No | Not reduce effect | No | No | Very low |
|  | CMV | 8 | 0 | 0 | 6 | Serious | No serious | Serious | Serious | No | Not reduce effect | No | No | Very low |
|  | Cpn | 11 | 0 | 0 | 11 | Serious | Serious | No serious | Serious | No | Not reduce effect | No | No | Very low |
| Kim, 2017 | peripheral blood BDNF | 20 | 0 | 0 | 20 | No serious | No serious | No serious | No serious | No | Not reduce effect | No | No | Low |
| Ou, 2020 | midlife hypertension | 4 | 0 | 4 | 0 | Serious | No serious | No serious | No serious | No | Not reduce effect | No | No | Very low |
|  | late-life hypertension | 18 | 0 | 18 | 0 | Serious | No serious | No serious | No serious | No | Not reduce effect | No | No | Very low |
|  | AHMs | 12 | 0 | 12 | 0 | Serious | No serious | No serious | No serious | No | Not reduce effect | No | No | Very low |
| Wang, 2015 | all NSAIDs | 16 | 0 | 12 | 4 | Serious | No serious | No serious | No serious | No | Not reduce effect | No | No | Very low |
|  | aspirin | 11 | 0 | 8 | 3 | Serious | No serious | No serious | No serious | No | Not reduce effect | No | No | Very low |
|  | no-aspirin NSAIDs | 8 | 0 | 7 | 1 | Serious | Serious | No serious | No serious | No | Not reduce effect | No | No | Very low |
| Shen, 2015 | Homocysteine | 9 | 0 | 0 | 9 | No serious | Serious | No serious | No serious | No | Not reduce effect | No | No | Very low |
|  | Folic Acid | 6 | 0 | 0 | 6 | No serious | Serious | No serious | No serious | No | Not reduce effect | No | No | Very low |
| Kunutsor, 2022 | serum uric acid | 20 | 0 | 0 | 21 | No serious | Serious | No serious | No serious | No | Not reduce effect | No | No | Very low |
| Luo, 2022 | Metformin | 10 | 0 | 5 | 5 | No serious | No serious | No serious | No serious | No | Not reduce effect | No | No | Low |
| Liu, 2022 | IBD | 5 | 0 | 4 | 1 | No serious | Serious | Serious | Serious | NA | Not reduce effect | No | No | Very low |
| Chai, 2019 | VD | 6 | 0 | 5 | 1 | No serious | No serious | No serious | Serious | No | Not reduce effect | No | No | Very low |
| Fan, 2019 | Long Sleep | 6 | 0 | 6 | 0 | No serious | No serious | No serious | Serious | No | Not reduce effect | No | No | Very low |
|  | Short Sleep | 6 | 0 | 6 | 0 | No serious | No serious | No serious | Serious | No | Not reduce effect | No | No | Very low |
| Li, 2017 | Cu in serum | 35 | 0 | 0 | 35 | No serious | Serious | No serious | Serious | No | Not reduce effect | No | No | Very low |
|  | Zn in serum | 22 | 0 | 0 | 22 | No serious | Serious | No serious | No serious | No | Not reduce effect | No | No | Very low |
|  | Fe in serum | 25 | 0 | 0 | 25 | No serious | Serious | No serious | No serious | No | Not reduce effect | No | No | Very low |
| Lee, 2020 | GA | 17 | 0 | 3 | 14 | Serious | No serious | No serious | No serious | No | Not reduce effect | No | No | Very low |
| Jalilian, 2018 | extremely low frequency magnetic fields | 20 | 0 | 5 | 15 | No serious | No serious | No serious | No serious | Strongly suspected | Not reduce effect | No | No | Very low |
| Dhiman, 2022 | PM2.5 | 6 | 0 | 6 | 0 | Serious | Serious | No serious | No serious | Strongly suspected | Not reduce effect | No | No | Very low |
|  | O3 | 4 | 0 | 4 | 0 | Serious | Serious | No serious | No serious | Strongly suspected | Not reduce effect | No | No | Very low |
| Qu, 2021 | plasma/serum β-carotene levels | 14 | 0 | 4 | 10 | No serious | Serious | No serious | No serious | Strongly suspected | Not reduce effect | No | No | Very low |
|  | plasma/serum α-carotene levels | 10 | 0 | 3 | 7 | No serious | Serious | No serious | Serious | Strongly suspected | Not reduce effect | No | No | Very low |
|  | plasma/serum lycopene | 10 | 0 | 2 | 8 | No serious | Serious | No serious | Serious | No | Not reduce effect | No | No | Very low |
|  | plasma/serum lutein levels | 10 | 0 | 3 | 7 | No serious | Serious | No serious | Serious | Strongly suspected | Not reduce effect | No | No | Very low |
|  | plasma/serum β-cryptoxanthin | 9 | 0 | 2 | 7 | No serious | Serious | No serious | Serious | Strongly suspected | Not reduce effect | No | No | Very low |
|  | plasma/serum zeaxanthin | 10 | 0 | 3 | 7 | No serious | Serious | No serious | Serious | Strongly suspected | Not reduce effect | No | No | Very low |
| Zhu, 2021 | DHA | 3 | 0 | 3 | 0 | No serious | Serious | No serious | No serious | NA | Not reduce effect | No | No | Very low |
|  | EPA | 3 | 0 | 3 | 0 | No serious | No serious | No serious | No serious | NA | Not reduce effect | No | No | Very low |
| Lv, 2016 | low testosterone | 7 | 0 | 7 | 0 | No serious | No serious | No serious | Serious | No | Not reduce effect | No | No | Very low |
| Gudala, 2013 | diabetes | 20 | 0 | 20 | 0 | No serious | No serious | No serious | No serious | No | Not reduce effect | No | No | Low |
| Shi, 2018 | sleep disturbances | 9 | 0 | 9 | 0 | Serious | No serious | No serious | No serious | No | Not reduce effect | No | No | Very low |
| Tsai, 2023 | AMD | 6 | 0 | 6 | 0 | No serious | No serious | No serious | No serious | Strongly suspected | Not reduce effect | No | No | Very low |
| Ahn, 2023 | PPI | 5 | 0 | 5 | 0 | No serious | Serious | Serious | No serious | No | Not reduce effect | No | No | Very low |
| Li, 2023 | Tooth loss | 6 | 0 | 6 | 0 | No serious | Serious | No serious | Serious | No | Not reduce effect | No | No | Very low |
| Xiong, 2023 | Cataract | 9 | 0 | 8 | 1 | Serious | No serious | No serious | No serious | Strongly suspected | Not reduce effect | No | No | Very low |
| Zhao, 2023 | Antioxidants | 12 | 0 | 12 | 0 | Serious | No serious | No serious | No serious | No | Not reduce effect | No | No | Very low |
| Schliep, 2023 | HDP | 3 | 0 | 3 | 0 | No serious | No serious | No serious | No serious | NA | Not reduce effect | No | No | Low |
| Yu, 2019 | Stroke | 7 | 0 | 7 | 0 | Serious | No serious | No serious | No serious | No | Not reduce effect | No | No | Very low |
|  | Vitamin C | 6 | 0 | 6 | 0 | No serious | No serious | No serious | Serious | No | Not reduce effect | No | No | Very low |
|  | Cognitive activity | 6 | 0 | 6 | 0 | No serious | No serious | No serious | Serious | No | Not reduce effect | No | No | Very low |
|  | High education | 7 | 0 | 7 | 0 | Serious | Serious | No serious | No serious | No | Not reduce effect | No | No | Very low |
| Qiao, 2022 | Loneliness | 3 | 0 | 3 | 0 | Serious | No serious | No serious | Serious | NA | Not reduce effect | No | No | Very low |

TC: total cholesterol, TG: total triglycerides, HDL-C: high-density lipoprotein, LDL-C: low-density lipoprotein, CHD: coronary heart disease, ApoA-I: Napoli protein A1, BMI: body mass index, RA: rheumatoid arthritis, BMD: bone mineral density, HSV-1: herpes simplex virus, CMV: cytomegalovirus, Cpn: chlamydia pneumonia, AHMs: anti-hypertensive medications, NSAIDs: non-steroidal anti-inflammatory drugs, IBD: inflammatory bowel disease, VD: vitamin D, GA: general anesthesia, PM_2.5_: particulate matter, O_3_: ozone, DHA: docosahexaenoic acid, EPA: eicosapentaenoic acid, AMD: age-related macular degeneration, PPI: proton pump inhibitors, HDP: Hypertensive Disorders of Pregnancy

NA: not available

**Supplementary Table 6** Assessment of 84 meta-analyses of the 53 articles about risk factors with Alzheimer's disease

| **Risk factor** | **sample cases** | **Random effects (95% CI)** | **P-value threshold (random effect model)** | **95% PI** | **Small study effect/excess significance bias** | **Heterogeneity estimates** |
| --- | --- | --- | --- | --- | --- | --- |
| Class I (convincing evidence) | | | | | | |
| RA | >1000 | 0.60(0.49-0.72) | <10^-6^ | Excluding the null value | No/No | Not large |
| Diabetes | ＞1000 | 1.58(1.43-1.76) | <10^-6^ | Excluding the null value | No/No | Not large |
| Class II (highly suggestive evidence) | | | | | | |
| Depression | >1000 | 1.79(1.46-2.20) | <10^-6^ | Including the null value | No/Yes | Very large |
| High Homocysteine level | ＞1000 | 1.89(1.38-2.33) | <10^-6^ | Excluding the null value | Yes/No | Not large |
| Low Folic Acid level | ＞1000 | 2.22(1.71-2.89) | <10^-6^ | Including the null value | No/No | Not large |
| Class III (suggestive evidence) | | | | | | |
| Atrial fibrillation | >1000 | 1.30(1.11-1.47) | >10^-6^ but <0.001 | Including the null value | No/No | Very large |
| Vitamin E in the diet | >1000 | 0.77(0.68-0.87) | >10^-6^ but <0.001 | Excluding the null value | No/No | Not large |
| Atherosclerosis | >1000 | 1.50(1.24-1.80) | >10^-6^ but <0.001 | Including the null value | Yes/No | Very large |
| Exposure to aluminum | >1000 | 1.72(1.33-2.21) | >10^-6^ but <0.001 | Including the null value | No/No | Not large |
| Leisure activities | >1000 | 0.82(0.74-0.91) | >10^-6^ but <0.001 | Including the null value | Yes/No | Large |
| Statin | >1000 | 0.70(0.60-0.80) | >10^-6^ but <0.001 | Including the null value | Yes/No | Not large |
| Bone loss | >1000 | 1.81(1.28-2.55) | >10^-6^ but <0.001 | Including the null value | NE/No | Not large |
| Head injury | >1000 | 1.51(1.27-1.80) | >10^-6^ but <0.001 | Including the null value | No/No | Large |
| Epilepsy | >1000 | 2.24(1.39-3.59) | >10^-6^ but <0.001 | Including the null value | No/No | Very large |
| Midlife hypertension | ＞1000 | 1.19(1.08-1.32) | >10^-6^ but <0.001 | Excluding the null value | No/No | Not large |
| AHMs | ＞1000 | 0.81(0.72-0.91) | >10^-6^ but <0.001 | Including the null value | No/No | Large |
| All NSAIDs | ＞1000 | 0.70(0.57-0.85) | >10^-6^ but <0.001 | Including the null value | No/No | Large |
| Aspirin | ＞1000 | 0.77(0.64-0.93) | >0.001 but <0.05 | Including the null value | No/No | Large |
| Cu in serum | ＞1000 | 3.36(2.03-5.58) | >10^-6^ but <0.001 | Including the null value | No/No | Very large |
| Extremely low-frequency magnetic fields | ＞1000 | 1.72(1.37-2.15) | >10^-6^ but <0.001 | Excluding the null value | No/No | Large |
| Plasma/Serum zeaxanthin | >500 but <1000 | 2.95(1.53-5.59) | >0.001 but <0.05 | Including the null value | No/No | Very large |
| Sleep disturbances | ＞1000 | 1.70(1.24-2.33) | >0.001 but <0.05 | Including the null value | No/No | Very large |
| ApoA-I in serum | >500 but <1000 | 0.12(0.04-0.34) | >10^-6^ but <0.001 | Including the null value | No/No | Very large |
| No-aspirin NSAIDs | ＞1000 | 0.65(0.48-0.88) | >0.001 but <0.05 | Including the null value | No/No | Large |
| Cataract | >1000 | 1.17(1.08-1.27) | >10^-6^ but <0.001 | Including the null value | No/No | Not large |
| Antioxidants | >1000 | 0.83(0.74-0.92) | >10^-6^ but <0.001 | Including the null value | Yes/No | Not large |
| Class IV (weak evidence) | | | | | | |
| Current versus never smoking | >1000 | 1.41(1.11-1.80) | >0.001 but <0.05 | Including the null value | No/No | Large |
| Pesticide | >1000 | 1.34(1.08-1.67) | >0.001 but <0.05 | Excluding the null value | No/No | Not large |
| Alcohol consumption | >1000 | 0.67(0.50-0.92) | >0.001 but <0.05 | Including the null value | No/No | Very large |
| ApoA-I in plasma | <500 | 0.12(0.02-0.67) | >0.001 but <0.05 | Including the null value | No/No | Very large |
| TC | >1000 | 1.35(1.02-1.78) | >0.001 but <0.05 | Including the null value | No/Yes | Very large |
| Clusterin in CSF | <500 | 2.7(2.13-3.65) | <10^-6^ | Including the null value | NE/No | Not large |
| Headache | >1000 | 1.53(1.06-2.22) | >0.001 but <0.05 | Including the null value | No/No | Large |
| BMD | <500 | 0.11(0.03-0.38) | >10^-6^ but <0.001 | Including the null value | NE/No | Very large |
| Hearing loss | >1000 | 2.23(1.33-3.73) | >0.001 but <0.05 | Including the null value | No/No | Not large |
| Albuminuria | >500 but <1000 | 1.37(0.05-1.79) | >0.001 but <0.05 | Including the null value | Yes/No | Not large |
| HSV-1 | >500 but <1000 | 1.34(1.02-1.75) | >0.001 but <0.05 | Excluding the null value | No/Yes | Not large |
| CMV | <500 | 1.39(1.05-1.83) | >0.001 but <0.05 | Including the null value | No/No | Not large |
| Cpn | <500 | 4.56(1.59-13.05) | >0.001 but <0.05 | Including the null value | No/No | Large |
| Cancer | ＞1000 | 0.86(0.78-0.94) | >0.001 but <0.05 | Including the null value | No/No | Very large |
| Serum uric acid | ＞1000 | 3.86(1.48-10.05) | >0.001 but <0.05 | Including the null value | No/Yes | Very large |
| VD deficiency | ＞1000 | 1.36(1.13-1.65) | >0.001 but <0.05 | Including the null value | No/No | Large |
| Long Sleep | NR | 1.62(1.19-2.21) | >0.001 but <0.05 | Including the null value | No/No | Not large |
| Plasma/Serum lutein levels | >500 but <1000 | 3.81(1.97-7.39) | >10^-6^ but <0.001 | Including the null value | No/No | Very large |
| Low testosterone | <500 | 1.48(1.12-1.96) | >0.001 but <0.05 | Excluding the null value | No/No | Not large |
| AMD | ＞1000 | 1.21(1.02-1.44) | >0.001 but <0.05 | Including the null value | Yes/No | Large |
| Tooth loss | NR | 1.11(1.03-1.20) | >0.001 but <0.05 | Including the null value | Yes/No | Large |
| HDP | >1000 | 1.40(1.13-1.74) | >0.001 but <0.05 | Including the null value | NE/No | Not large |
| Stroke | >1000 | 1.38(1.02-1.88) | >0.001 but <0.05 | Including the null value | No/No | Not large |
| Vitamin C | >500 but <1000 | 0.84(0.71-1.00) | >0.001 but <0.05 | Including the null value | No/No | Not large |
| Cognitive activity | >500 but <1000 | 0.49(0.38-0.63) | <10^-6^ | Excluding the null value | No/No | Not large |
| Loneliness | <500 | 1.51(1.07-2.13) | >10^-6^ but <0.001 | Including the null value | NE/No | Not large |
| Non-significant evidence | | | | | | |
| Ever versus never smoking | >1000 | 1.12(0.99-1.26) | >0.05 | Including the null value | Yes/No | Large |
| CHD | >1000 | 1.09(0.90-1.32) | >0.05 | Including the null value | Yes/No | Not large |
| Heart failure | >1000 | 1.41(0.98-2.03) | >0.05 | Including the null value | Yes/No | Large |
| Socioeconomic status | >1000 | 1.19(0.70-2.03) | >0.05 | Including the null value | Yes/No | Very large |
| ApoA-I in CSF | <500 | 1.43(0.74-2.76) | >0.05 | Including the null value | No/No | Large |
| HDL-C | >1000 | 0.76(0.54-1.1) | >0.05 | Including the null value | No/Yes | Very large |
| LDL-C | >1000 | 1.39(1.03-2.00) | >0.05 | Including the null value | No/No | Very large |
| TG | >1000 | 1.29(1.12-1.85) | >0.05 | Including the null value | No/Yes | Very large |
| Clusterin in plasma | >1000 | 1.41(1.19-2.39) | >0.05 | Including the null value | No/No | Very large |
| Underweight | >1000 | 1.43(0.86-2.39) | >0.05 | Including the null value | No/No | Large |
| Overweight | >1000 | 1.02(0.80-1.30) | >0.05 | Including the null value | No/No | Very large |
| Obese | >1000 | 1.20(0.76-1.88) | >0.05 | Including the null value | No/No | Very large |
| Caffeine | >500 but <1000 | 0.79(0.49-1.27) | >0.05 | Including the null value | No/No | Large |
| Frailty | >1000 | 1.28(0.88-1.85) | >0.05 | Including the null value | No/No | Very large |
| Peripheral blood BDNF | ＞1000 | 0.74(0.48-1.15) | >0.05 | Including the null value | No/Yes | Very large |
| Late-life hypertension | ＞1000 | 0.94(0.85-1.05) | >0.05 | Including the null value | Yes/Yes | Not large |
| Metformin | >1000 | 1.15(0.82-1.63) | >0.05 | Including the null value | No/No | Very large |
| IBD | NR | 1.65(0.84-3.26) | >0.05 | Including the null value | Yes/No | Very large |
| Zn in serum | ＞1000 | 0.62(0.30-1.27) | >0.05 | Including the null value | No/Yes | Very large |
| Fe in serum | ＞1000 | 0.81(0.40-1.67) | >0.05 | Including the null value | No/Yes | Very large |
| GA | NR | 0.92(0.82-1.05) | >0.05 | Including the null value | No/No | Not large |
| Short Sleep | NR | 1.19(0.91-1.56) | >0.05 | Including the null value | No/No | Large |
| PM2.5 | ＞1000 | 1.08(0.98-1.18) | >0.05 | Including the null value | No/No | Very large |
| O3 | ＞1000 | 1.02(0.97-1.07) | >0.05 | Including the null value | No/No | Very large |
| Plasma/Serum β-carotene levels | ＞1000 | 0.96(0.52-1.80) | >0.05 | Including the null value | No/No | Very large |
| Plasma/Serum α-carotene levels | >500 but <1000 | 0.69(0.32-1.49) | >0.05 | Including the null value | No/Yes | Very large |
| Plasma/Serum lycopene | >500 but <1000 | 0.80(0.15-4.29) | >0.05 | Including the null value | No/Yes | Very large |
| Plasma/Serum β-cryptoxanthin | >500 but <1000 | 0.85(0.21-3.46) | >0.05 | Including the null value | No/Yes | Very large |
| DHA | ＞1000 | 0.75(0.42-1.34) | >0.05 | Including the null value | NE/No | Large |
| EPA | ＞1000 | 0.91(0.66-1.25) | >0.05 | Including the null value | NE/No | Large |
| PPI | ＞1000 | 1.15(0.95-1.40) | >0.05 | Including the null value | No/No | Very large |
| High education | ＞1000 | 0.95(0.89-1.00) | >0.05 | Including the null value | No/No | Large |

TC: total cholesterol, TG: total triglycerides, HDL-C: high-density lipoprotein, LDL-C: low-density lipoprotein, CHD: coronary heart disease,

ApoA-I: Napoli protein A1, BMI: body mass index, RA: rheumatoid arthritis, BMD: bone mineral density, HSV-1: herpes simplex virus, CMV: cytomegalovirus, Cpn: chlamydia pneumonia, AHMs: anti-hypertensive medications, NSAIDs: non-steroidal anti-inflammatory drugs, IBD: inflammatory bowel disease, VD: vitamin D, GA: general anesthesia, PM_2.5_: particulate matter, O_3_: ozone, DHA: docosahexaenoic acid, EPA: eicosapentaenoic acid, AMD: age-related macular degeneration, PPI: proton pump inhibitors, HDP: Hypertensive Disorders of Pregnancy

NE: not estimable because less than three studies were available
